# Effects of a Comprehensive Dietary Intervention Program, Promoting Nutrition Literacy, Eating Behavior, Dietary Quality, and Gestational Weight Gain in Chinese Urban Women with Normal Body Mass Index during Pregnancy

**DOI:** 10.3390/nu16020217

**Published:** 2024-01-10

**Authors:** Qian Li, Noppawan Piaseu, Srisamorn Phumonsakul, Streerut Thadakant

**Affiliations:** 1 Ramathibodi School of Nursing, Faculty of Medicine Ramathibodi Hospital, Mahidol University, 270 Rama 6 Road, Ratchathewi, Bangkok 10400, Thailand; liqian_2021@126.com (Q.L.); srisamorn.phu@mahidol.ac.th (S.P.); streerut.bor@mahidol.edu (S.T.); 2 Ph.D. Candidate in the Doctor of Philosophy Program in Nursing Science (International Program), Faculty of Medicine Ramathibodi Hospital, Faculty of Nursing, Mahidol University, Salaya 73170, Thailand

**Keywords:** pregnancy, health literacy, eating behavior, weight, nutrition, digital, intervention

## Abstract

In urban Chinese women with normal body weight during pregnancy, we implemented a comprehensive dietary intervention program aimed at enhancing nutrition literacy, dietary quality, and gestational weight gain. The methods included both online and offline health education on prenatal nutrition, weekly weight monitoring, family back education practices, and real-time dietary guidance. The intervention was delivered to randomly assigned control and intervention group participants from gestational week 12 to week 24. The intervention group (*n* = 44; 100% complete data) showed significant differences (mean (SD)) compared to the control group (*n* = 42; 95.5% complete data) in nutrition literacy (53.39 ± 6.60 vs. 43.55 ± 9.58, *p* < 0.001), restrained eating (31.61 ± 7.28 vs. 28.79 ± 7.96, *p* < 0.001), Diet Quality Distance (29.11 ± 8.52 vs. 40.71 ± 7.39, *p* < 0.001), and weight gain within the first 12 weeks of intervention (4.97 ± 1.33 vs. 5.98 ± 2.78, *p* = 0.029). However, there was no significant difference in the incidence of gestational diabetes (2 (4.5%) vs. 4 (9.5%), *p* = 0.629). Participants in the intervention group reported an overall satisfaction score of 4.70 ± 0.46 for the intervention strategy. These results emphasize the positive role of comprehensive dietary intervention in promoting a healthy diet during pregnancy.

## 1. Introduction

Maternal and child health are crucial determinants of a nation’s overall health and development. The impact of weight gain during pregnancy on short-term and long-term health outcomes underscores its significance in comprehensive pregnancy health management [1,2,3,4]. The findings, based on existing research evidence, suggested that excessive gestational weight gain (GWG) could lead to cesarean section, maternal weight retention, large-for-gestational-age (LGA) infants, gestational hypertension, preeclampsia, and gestational diabetes mellitus (GDM). On the other hand, insufficient GWG was associated with a higher risk of small-for-gestational-age (SGA) infants and preterm birth (PTB).

In a study by Hu that included 1260 Chinese pregnant women, it was found that 60.4% had a normal weight before pregnancy, 19.44% were overweight, and 6.98% were obese based on BMI classification. When comparing their weight gain during pregnancy to the reference values recommended by the American Institute of Medicine (IOM) guidelines in 2009, it was observed that 54.97% had excessive GWG, 34.65% had appropriate GWG, and 10.38% had insufficient GWG [5]. Similar findings were reported in several other surveys conducted on pregnant women in China, indicating that most pregnant women did not meet the weight-gain recommendations outlined by the IOM [6,7]. These statistics highlight the need for attention and intervention to address the issue of inappropriate GWG in China.

GWG, as an indicator of nutritional balance during pregnancy, is closely associated with individual dietary behaviors. While physical activity during pregnancy is acknowledged as a factor influencing GWG, systematic reviews suggest that diet may play a more significant role in determining weight gain during pregnancy [8,9]. Therefore, promoting dietary behaviors during pregnancy becomes crucial for enhancing the health of pregnant women. However, over time, many studies on dietary interventions during pregnancy have yielded mixed results, particularly a significant portion of online dietary intervention studies show no effect [10,11,12,13,14]. This may be related to insufficient key components in the intervention design, which may not have effectively addressed the critical barriers in pregnant women’s health dietary practices [15].

Several qualitative studies conducted from the perspective of pregnant women have shed light on the substantial impediments they encounter in their dietary practices [16,17,18,19,20,21,22,23,24,25,26,27,28,29,30,31,32,33,34,35,36,37,38,39,40,41]. These obstacles primarily revolve around deficiencies in the dietary information delivery system, encompassing challenges related to accessing reliable dietary information; cognitive aspects such as understanding, memorization, and the application of dietary information; as well as the skills required to effectively communicate dietary information within the family environment. These findings underscore the pervasive issue of nutritional literacy deficiency among pregnant women [42].

This is in contrast to previous studies that have shown ineffective outcomes, particularly those focusing on online dietary interventions [10,11,12,13,14], which mainly provide static text or video information with limited incorporation of interactive information consultations or regular text responses. However, the acquisition of dietary information does not inherently impart healthy dietary knowledge and skills. Furthermore, it does not imply that pregnant women possess the necessary capacity for healthy self-care. Information acquisition merely marks the beginning of this process [43]. Therefore, researchers should emphasize assessing the proficiency of pregnant women in processing and applying the received information. However, to the best of our knowledge, there is still a lack of research investigating the influence of maternal nutritional literacy on self-care behaviors related to diet during pregnancy.

Digital platforms, with features such as wide accessibility, personalized services, interactivity, real-time updates, and cost-effectiveness, have become ideal tools for implementing comprehensive health education and enhancing health literacy [44]. Nutbeam’s health literacy model indicates that health literacy can be effectively improved through tailored information, communication, and education [45].

Therefore, guided by the health literacy model, this study aimed to address the overlooked aspect of maternal health literacy in the provision of general dietary information online. To achieve this goal, an online platform was used to implement a tailored, ongoing, face-to-face health education program. The primary objective was to design a comprehensive, individual-level dietary intervention program that would offer pregnant women easy access to information and help improve their nutritional literacy. By enhancing the dietary behavior of pregnant women in urban China, the study sought to elevate their overall diet quality and nutritional status, ultimately reducing the risk of pregnancy complications.

## 2. Materials and Methods

### 2.1. Study Design

This study adopts a two-arm randomized controlled trial design, employing a prospective and pragmatic implementation approach. The variable positioning is based on Orem’s self-care theory [46], and the intervention design is grounded in Nutbean’s conceptual model of health literacy as an asset [45]. The report follows the CONSORT framework. Approval was obtained from the Human Research Ethics Committee, Faculty of Medicine Ramathibodi Hospital, Mahidol University (MURA2023/590), and the Clinical Research Ethics Committee of the Affiliated Changzhou No. 2. People’s Hospital of Nanjing Medical University ([2023]KY107-01). All methods adhere to relevant guidelines and regulations, such as the Helsinki Declaration. The trial has been registered with the China Clinical Trial Registration Center (ChiCTR2300075082).

### 2.2. Participants and Recruitment

This study was conducted at an Obstetrics Outpatient Department in Changzhou, China, from August to November 2023. Through systematic sampling based on clinic serial numbers, pregnant women were initially screened by obstetricians, and those in their 6–12 weeks of gestation and primiparous were referred to the eligibility assessment room. Trained midwives conducted a thorough eligibility screening. Inclusion criteria comprised age between 18 and 35, pre-pregnancy BMI between 18.5 kg/m² and 24 kg/m², primiparous individuals with a single pregnancy, and gestational age less than 12 weeks. Participants needed the ability to use the WeChat application, and households had to include at least one person other than the pregnant woman who served as a cook. Exclusion criteria included various health conditions and behaviors such as diabetes, uncontrolled high blood pressure, thyroid disease, cardiovascular disease, cancer, lung disease, severe gastrointestinal disease, a history of eating disorders or bariatric surgery, serious mental illness, a history of mood and anxiety disorders in the last three months, drug abuse, and a threat of abortion. Participants were withdrawn if they experienced illness or required special dietary needs during the intervention. Eligible pregnant women who provided consent and completed the baseline assessment were randomized into two groups, utilizing the SAS program for full randomization: usual care (n = 44) and Comprehensive Dietary Intervention Program (CDIP) groups (n = 44). The midwives conducting the assessments were unaware of the group assignments. Pregnant women in the control group received standard antenatal care common for Chinese pregnant women, consisting of regular prenatal check-ups and monitoring. Pregnant women in the intervention group received a CDIP intervention alongside routine care based on the standards from the Chinese Dietary Guidelines for Pregnancy and the GWG range recommended by the IOM.

### 2.3. Sample Size

Based on Deng’s randomized controlled trial [47], which reported a GWG of 6.9 ± 3.2 kg in the control group and 4.9 ± 3.1 kg in the intervention group, we determined that each group would require 40 women to achieve 80% power. Assuming a 10% dropout rate between baseline and follow-up, the planned recruitment target is set at 44 women per group.

### 2.4. Intervention—CDIP

Based on the theoretical foundation of the health literacy model, the CDIP intervention comprised three essential components: tailored information, communication, and education. Behavior change techniques (BCTs) corresponding to these structures were implemented [48], as outlined in Table 1. Following this, specific intervention topics and content were developed in accordance with the established BCTs. The intervention was implemented through a systematic and phased approach.

Phase 1: Offline Intervention

During the face-to-face consultation, participants underwent a 30–40 min session on the day of enrollment at 12 weeks of gestation. This consultation took place in the intervention room, covering topics 1–6. Instruments utilized included a diet booklet (see Figure S1) and food models.

Theme 1: Is your diet healthy? The intervention midwife analyzed baseline survey data with stakeholders, reviewing scores from the nutritional literacy scale to assess participants’ literacy levels and knowledge and skill deficiencies. The midwife also examined the intake of ten major nutrients using the diet quality scale, gaining insights into participants’ dietary control, food cravings, and home eating environment. The midwife documented individual eating problems and disorders.

Theme 2: What obstacles do you encounter? Participants confirmed the listed barriers, and solutions were discussed. These challenges pertain to difficulties in accessing, comprehending, identifying, and utilizing dietary information, as well as sharing such information with family members. Informing participants of the study schedule addressed these issues. Concerns about consultation time and cost were alleviated by informing participants of the study’s free and flexible online intervention. Participants with low self-efficacy were encouraged, and pregnant women lacking motivation were informed of the benefits of following guidelines during pregnancy, with a reward of a free fetal heart rate monitoring project upon completion.

Theme 3: Do you understand this information? Using the self-designed diet education booklet (see Figure S1), participants were introduced to “the Recommended Standards for Weight Gain During Pregnancy for Chinese Women [49]” and “the Chinese Dietary Guidelines During Pregnancy 2022 [50]”. They learned how to choose appropriate foods based on preferences, handle food cravings, and self-monitor body weight. Participants were asked to confirm their understanding and provide in-depth clarification if needed.

Theme 4: How is the meal plan implemented? Food models demonstrated daily food calculations based on dietary preferences and Chinese Dietary Guidelines for Pregnancy. This included substituting low-calorie for high-calorie foods, replacing expensive foods with low-cost alternatives, and methods for self-monitoring shared with health providers via WeChat.

Theme 5: Do you believe in yourself? We will help you! Participants were informed that subsequent CDIP interventions would be online, emphasizing convenience and low cost. This included watching pregnancy diet education videos, a family education exercise, weekly weight and meal quality measurement reports, and online discussions. Pregnant women were encouraged to adhere to the schedule, maintaining open communication with investigators.

Theme 6: Enjoy free items! Participants were informed about incentives, such as free fetal heart rate monitoring and continuous online/offline dietary counseling, by following the online intervention plan.

Phase 2: Online Intervention

Delivery was through the WeChat platform, with participants using a communication window for reminders and simultaneous diet education video viewing. The online program included twice-repeated diet education video sessions lasting 35 min (weeks 13 and 20), a 20–30 min return education exercise for family members (week 16), and two 20–30 min in-person meal-planning discussions (weeks 17 and 21). Participants also received a weekly weight-monitoring feedback text message service (SMS).

Theme 7: Did you do it today? During weeks 13 and 20, participants received reminders through WeChat to watch a dietary education video; the content of the dietary education video involves in-depth verbal explanations of the contents found in the dietary education booklet. Focusing on understanding and applying guidelines for pregnant women in China and GWG guidelines, the video explained how to manage energy intake and food cravings.

Theme 8: Pass the knowledge on to your family! At 16 weeks, pregnant women and home cooks met online. Pregnant women explained dietary information to their families based on an educational video. Health providers assessed accuracy, clarifying misconceptions to ensure understanding and utilization.

Theme 9: Let us see your progress! After monitoring food intake at weeks 16 and 20, participants engaged in online meal-planning discussions at weeks 17 and 21. The workshop included summary feedback, analyzing achievement against GWG guidelines, comparing food intake with dietary guidelines, and encouraging participants throughout the process.

Theme 10: What could you do next? The second part of the online discussion focused on individualized meal-plan adjustments based on participants’ weight-gain goals. The health provider discussed specific barrier factors and provided solutions. Food frequency measurements at weeks 17 and 21 informed dietary quality adjustments. The health provider advised on changes, maintained the meal plan when aligned with weight-gain goals, and offered encouragement at the consultation’s end.

### 2.5. Compensation

Following the completion of the baseline survey, all enrolled participants received an exquisite photo album and a gift bag (recommended retail value of 80 RMB). Additionally, pregnant women in the CDIP intervention group were provided with complimentary electronic fetal monitoring vouchers (valued at 200 RMB).

### 2.6. Variables Measures and Measurement Instruments

To achieve the goal of improving dietary behaviors among pregnant women in urban China, a comprehensive assessment of variables based on Orem’s self-care theory is planned [46]. Specifically, we are interested in self-care agency, self-care behavior, nutritional status, and relevant pregnancy complications during the process of maternal dietary self-management. Corresponding variables include nutritional literacy, eating behavior, dietary quality, GWG, and the incidence of gestational diabetes.

It is worth noting that this study is an individual-level dietary intervention project, and research suggests that family functioning and physical activity levels may influence maternal dietary behaviors. Therefore, at baseline, this study also measures these two variables to further assess their impact on the outcomes.

The Demographic Questionnaire serves to collect participants’ demographic information and comprises two sections—personal characteristics and sociocultural factors—with a total of 8 items. Inquiries encompass age, education level, gestational weeks, pre-pregnancy BMI, family average annual income, ethnic group, religion, cuisine, and family structure.

The Pregnancy Physical Activity Questionnaire in Chinese (PPAQ-C) is employed to assess the baseline physical activity levels of study subjects [51]. In this measurement, participants report their pregnancy activity levels over the past two weeks, covering aspects such as household chores, outdoor activities, occupational tasks, and exercise, totaling 31 items. Through participants’ responses to each item, including activity duration and corresponding energy expenditure values, we can calculate the baseline pregnancy activity.

The APGAR questionnaire is utilized to evaluate family functioning, including five aspects: Adaptation, Partnership, Growth, Affection, and Resolve [52]. Each aspect is graded on three levels: “2 points for ‘often’”, “1 for ‘sometimes’”, and “0 for ‘rarely’”. The total score ranges from 0 to 10 points, with higher scores indicating better family functioning.

The Nutrition Literacy Assessment Instrument for Pregnant Women in China (NLAI-P) is employed to measure participants’ nutritional literacy [53]. Participants respond to 38 questions across three dimensions: knowledge literacy, behavior literacy, and skill literacy. Scores for each dimension and the total score are calculated based on the scoring criteria provided by the instrument developer, with higher scores indicating higher levels of nutritional literacy during pregnancy.

The Dutch Eating Behavior Questionnaire—Chinese version (DEBQ-C) is used to assess eating behavior [54,55], with participants quickly responding to 33 questions covering three subscales: restrained eating, emotional eating, and external eating. Scores for each subscale and the total score are separately calculated, and higher scores reflect higher levels of eating behavior in the respective dimensions.

The Food Frequency Questionnaire for Pregnant Women (FFQ-P) assesses participants’ dietary quality by asking them to recall their dietary habits over the past four weeks [56]. The questionnaire includes 61 food items grouped into ten categories such as meat, fish, vegetables, and fruits. We compare it to the Chinese Diet Balance Index for Pregnancy (DBI-P) to calculate participants’ dietary balance index [57]. Details of the DBI scoring are described elsewhere [58,59]. We compute balance coefficients for each food category and the overall dietary quality distance. A score closer to 0 indicates a more balanced diet, while negative distances suggest more severe underconsumption, and positive distances indicate more severe overconsumption.

Assessors in the hospital conducted pre- and post-weight measurements using a standard weight scale. We calculated the weight gain during the twelve-week intervention period (from week 12 to week 24 of pregnancy).

Gestational diabetes mellitus (GDM) diagnosis: GDM diagnosis is based on blood glucose levels obtained from the 75 g Oral Glucose Tolerance Test (OGTT) conducted at 24 weeks of pregnancy, following the diagnostic criteria established by the International Association of Diabetes and Pregnancy Study Group (IADPSG) [60].

### 2.7. Analysis

Quantitative data analysis was performed using IBM SPSS Statistics v28 (IBM Corp, Armonk, New York, NY, USA). Basic statistics, including the calculation of mean and standard deviation (for normal distribution), median and interquartile range (IQR) (for skewed distribution), as well as frequency and percentage, were conducted. When comparing baseline data, an independent samples *t*-test was employed if continuous measurement variables met the assumption of normality. Otherwise, the Mann–Whitney U test or Chi-squared test was used to examine differences. For within-group comparisons before and after interventions, a paired samples *t*-test was applied if the differences in continuous measurement variables met the assumption of normality; otherwise, the Paired Wilcoxon Signed Ranks Test was used. In between-group comparisons after interventions, if continuous variables met the assumptions of normal distribution and homogeneity of variances, the One-way analysis of covariance was used, with baseline data as covariates; otherwise, the Mann–Whitney U test was utilized. For frequency data results with more than 20% cell counts less than the minimum expected count, a Fisher’s Chi-squared test was performed. Intergroup comparisons were used to validate research hypotheses, and *p*-values were obtained using a one-tailed test. In all comparative analyses, *p* < 0.05 was considered statistically significant. Statistical analysis will adhere to the principles of intention-to-treat analysis, and missing data values will be handled using Complete-case analysis [61].

## 3. Results

### 3.1. Study Implementation

In this study, the trial was registered with the China Clinical Trial Registration Center under registration number ChiCTR2300075082. This investigation was carried out from August 2023 to November 2023. A total of 2712 individuals were systematically sampled from the Obstetrics Outpatient Department of a tertiary healthcare facility located in Changzhou, China. Among the 88 participants who met the specified inclusion and exclusion criteria and demonstrated a voluntary commitment to participation, they were subjected to a random allocation process, segregating them into two distinct groups, with each cohort comprising 44 individuals.

In the CDIP group, no participants were withdrawn, and compliance and retention were excellent, with no instances of participant attrition. Conversely, the routine care group experienced the attrition of two participants, with one exiting the study due to high-risk pregnancy complications and the other as a result of discontinued communication. Consequently, post-intervention data were acquired from the remaining 42 participants.

**Figure 1:**
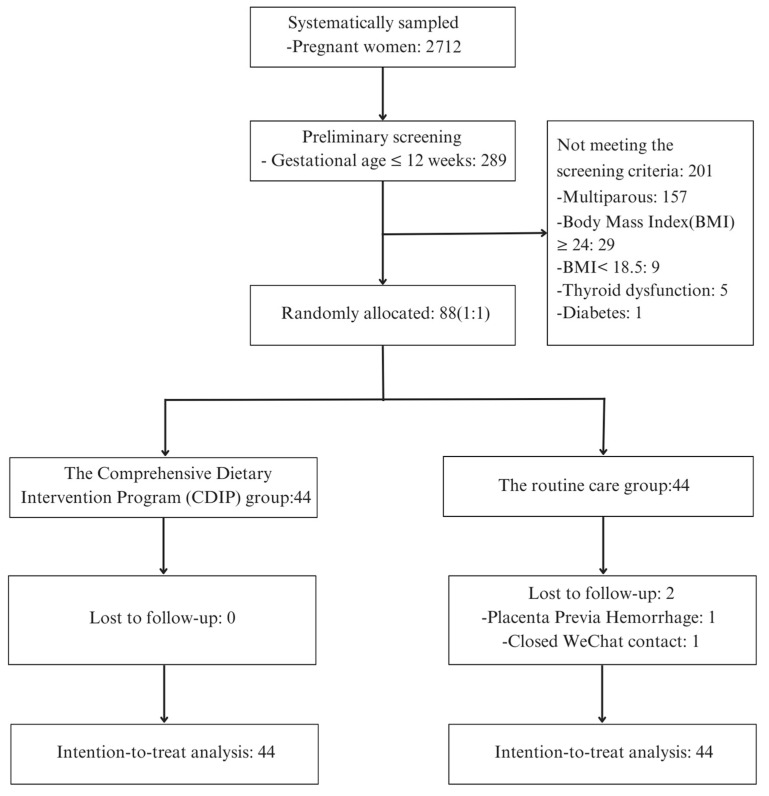
Recruitment and intervention flowchart.

Consistent with the principles of intention-to-treat analysis, data from all 88 participants were inclusively considered in the subsequent analysis. A comprehensive recruitment and intervention process is visually depicted in Figure 1 for reference.

### 3.2. Comparison of General Characteristics of Study Participants before Intervention

The average age of participants was (26.51 ± 2.96) years, and the two groups showed no statistically significant differences in terms of age, education level, family annual income, and cuisine preference. The average gestational age at baseline was (11.85 ± 0.41) weeks, and the pre-pregnancy Body Mass Index (BMI) was (21.14 ± 1.63) kg/m^2^. There were no statistically significant differences between the two groups in terms of gestational age at baseline, pre-pregnancy BMI, family function, and physical activity level indicators. For detailed information on the general characteristics of study participants, please refer to Table 2.

### 3.3. Baseline Comparison of Outcome Measures for Participants before Intervention

**Figure 2:**
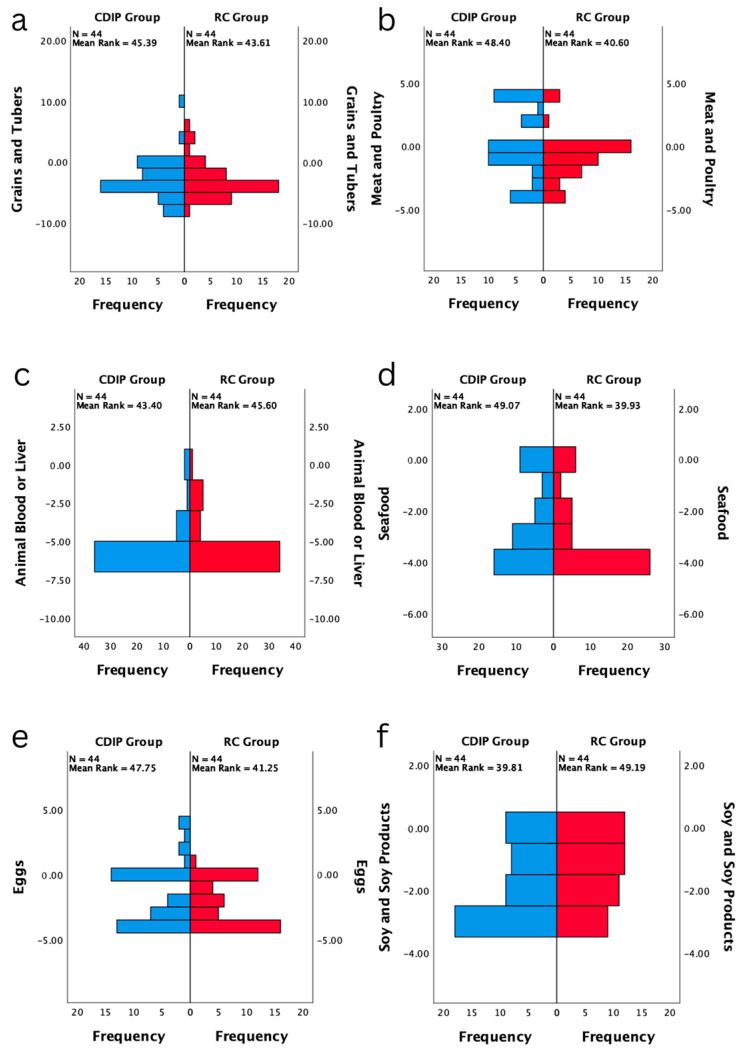

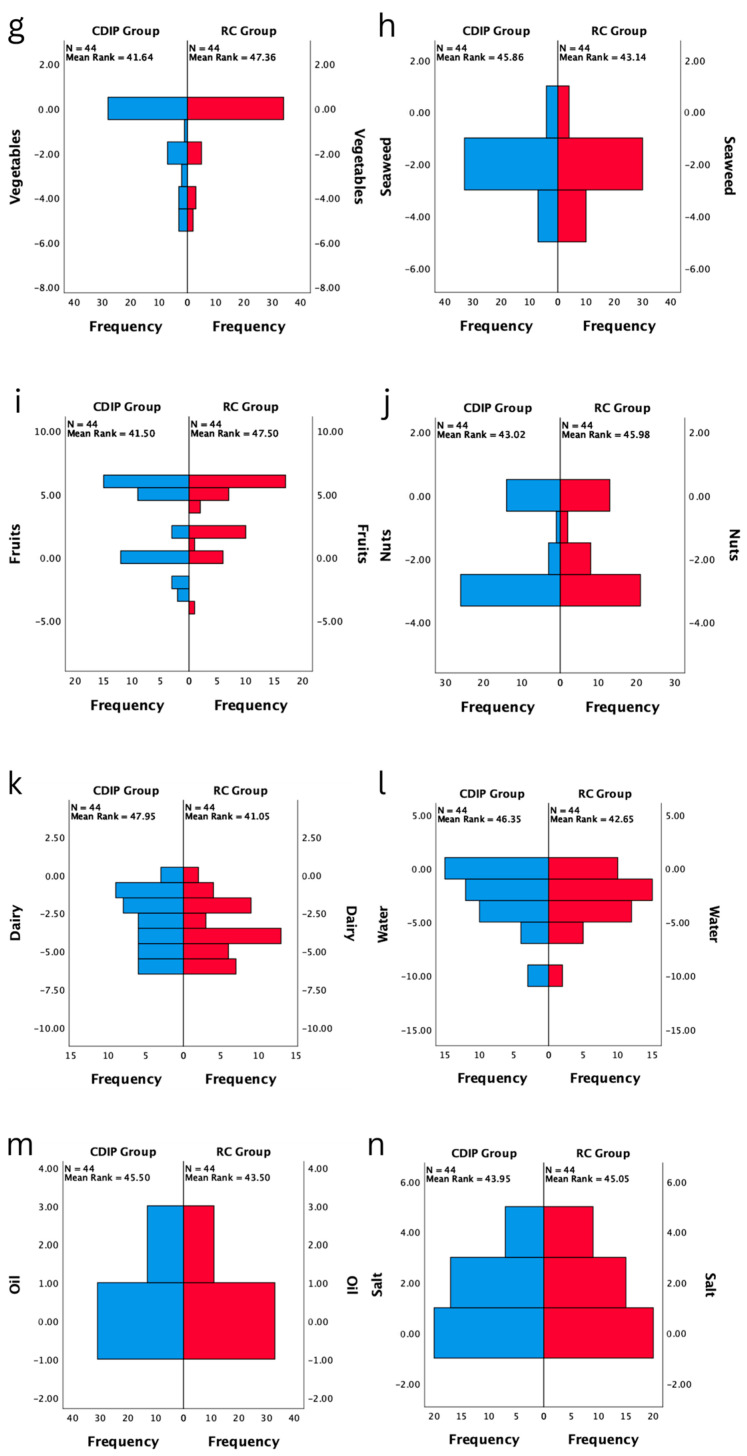
Baseline comparison of balance coefficients for various nutrients in two groups (*n* = 88). Note: (**a**) represents the comparison of frequency distributions in the dietary balance index of grains and tubers between two post-test groups; (**b**) signifies the comparison of frequency distributions in the dietary balance index of meat and poultry between two post-test groups; (**c**) denotes the comparison of frequency distributions in the dietary balance index of animal blood or liver between two post-test groups; (**d**) indicates the comparison of frequency distributions in the dietary balance index of seafood between two post-test groups; (**e**) stands for the comparison of frequency distributions in the dietary balance index of eggs between two post-test groups; (**f**) illustrates the comparison of frequency distributions in the dietary balance index of soy and soy products between two post-test groups; (**g**) showcases the comparison of frequency distributions in the dietary balance index of vegetables between two post-test groups; (**h**) highlights the comparison of frequency distributions in the dietary balance index of seaweed between two post-test groups; (**i**) focuses on the comparison of frequency distributions in the dietary balance index of fruits between two post-test groups; (**j**) portrays the comparison of frequency distributions in the dietary balance index of nuts between two post-test groups; (**k**) outlines the comparison of frequency distributions in the dietary balance index of dairy between two post-test groups; (**l**) emphasizes the comparison of frequency distributions in the dietary balance index of water between two post-test groups; (**m**) represents the comparison of frequency distributions in the dietary balance index of oil between two post-test groups; (**n**) signifies the comparison of frequency distributions in the dietary balance index of salt between two post-test groups.

After conducting statistical analysis on the two groups of study participants, it was found that there were no statistically significant differences between the two groups in terms of baseline prenatal nutritional literacy, eating behavior, dietary quality, and weight gain prior to the pre-test. Specific data can be found in Table 3. The dietary balance coefficients for various nutrient categories showed no statistically significant differences between the two groups. It was also observed that the median coefficients for dietary oil and vegetables were at a balanced zero point during the pre-test. Only the median coefficient for the fruit category was above zero, indicating excess intake, while the other categories were below zero, indicating inadequate intake. Refer to Figure 2 for details.

### 3.4. Impact of Intervention on Eating Behavior in Urban Chinese Pregnant Women

Intra-group comparison results show that pregnant women receiving CDIP intervention demonstrated a significant improvement in total eating behavior scores and restrained eating dimension scores compared to baseline (*p* < 0.05). In contrast, pregnant women receiving routine care intervention showed no statistically significant differences in scores compared to baseline. Detailed results are presented in Table 4.

Inter-group comparison results, after adjusting for pre-intervention eating behavior score levels, indicate that following intervention, the total eating behavior score for the CDIP group was significantly higher than the routine care group by an average of 3.87 points (95% CI: 0.336–7.395, *p* = 0.032). Similarly, the restrained eating dimension score for the CDIP group after the intervention was, on average, 3.22 points higher than the routine care group, with a statistically significant difference (95% CI: 1.665–4.768, *p* < 0.001).

**Figure 3:**
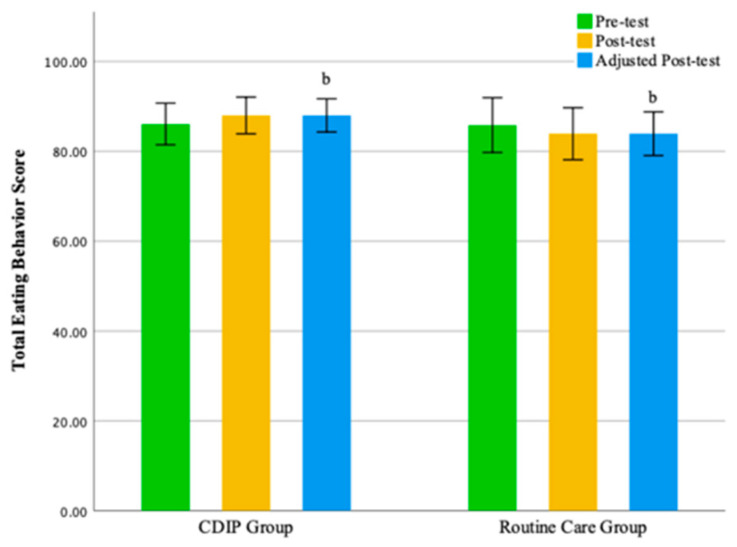
Illustrates the total eating behavior score before and after intervention in two groups. Note: CDIP is the Comprehensive Dietary Intervention Program, ‘b’ signifies Comparison with the Control Group, *p* < 0.05.

**Figure 4:**
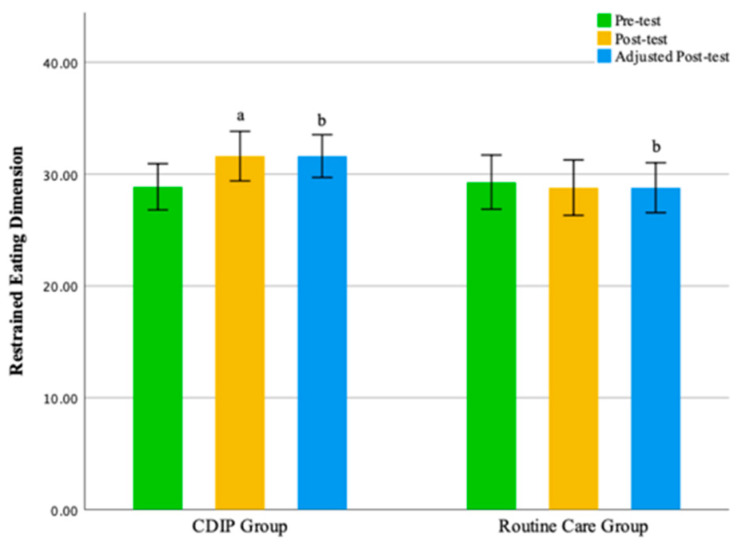
Illustrates the restrained eating dimension before and after intervention in two groups. Note: CDIP is the Comprehensive Dietary Intervention Program, ‘a’ denotes Intra-group Comparison, *p* < 0.05; ‘b’ signifies Comparison with the Control Group, *p* < 0.05.

**Figure 5:**
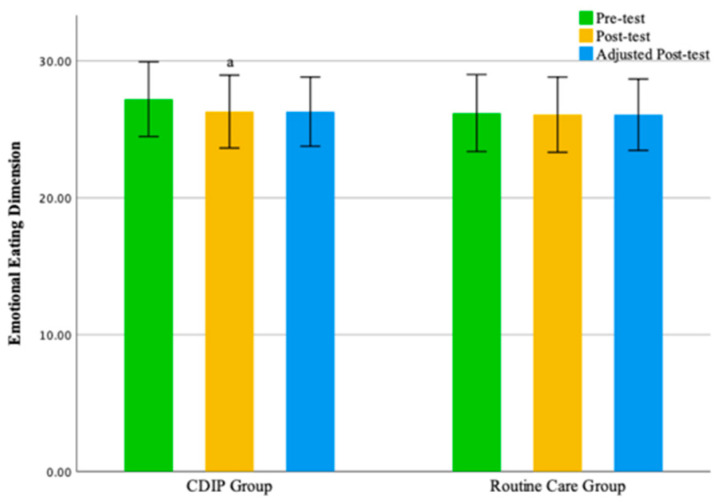
Illustrates the Emotional Eating Dimension Before and After Intervention in Two Groups. Note: CDIP is the Comprehensive Dietary Intervention Program, ‘a’ denotes Intra-group comparison, *p* < 0.05.

**Figure 6:**
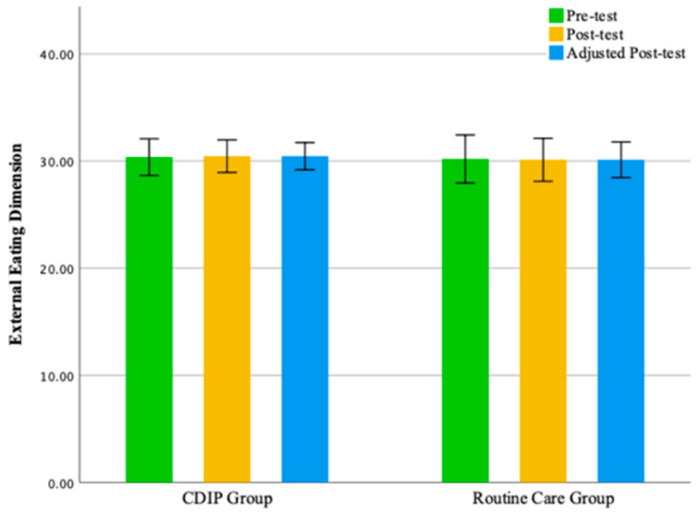
Illustrates the external eating dimension before and after intervention in two groups. Note: CDIP is the Comprehensive Dietary Intervention Program.

No statistically significant differences were observed between the two groups in the emotional eating dimension and external eating dimension. The findings of this study suggest that, compared to the control group, CDIP intervention contributes to an improvement in patients’ restrained eating behavior. Detailed results can be found in Table 5 and Table 6 as well as Figure 3, Figure 4, Figure 5 and Figure 6.

### 3.5. Impact of Intervention on Nutrition Literacy in Urban Chinese Pregnant Women

The intra-group comparison results indicate that pregnant women receiving CDIP intervention demonstrated significant improvements in Total Nutritional Literacy Score, Knowledge Literacy Dimension scores, Behavioral Literacy Dimension, and Skills Literacy Dimension compared to baseline (*p* < 0.05). In contrast, pregnant women receiving routine care intervention showed no statistically significant differences in scores compared to baseline. Detailed results are presented in Table 7.

The inter-group comparison results, after adjusting for pre-intervention nutritional literacy levels, reveal that following an intervention, the CDIP group’s overall gestational nutritional literacy score was significantly higher than the routine care group, averaging 9.64 points (95% CI: 8.445–10.836, *p* < 0.001). Similarly, the Knowledge Literacy Dimension score for the CDIP group after the intervention was, on average, 5.98 points higher than the routine care group, with a statistically significant difference (95% CI: 5.038–6.921, *p* < 0.001). The Behavioral Literacy Dimension score for the CDIP group after the intervention was, on average, 1.98 points higher than the routine care group, with a statistically significant difference (95% CI: 1.297–2.660, *p* < 0.001). Both groups’ Skills Literacy Dimension exhibited a non-normal distribution, with the CDIP group’s median surpassing that of the routine care group by 2.05 points after intervention (*p* = 0.001).

**Figure 7:**
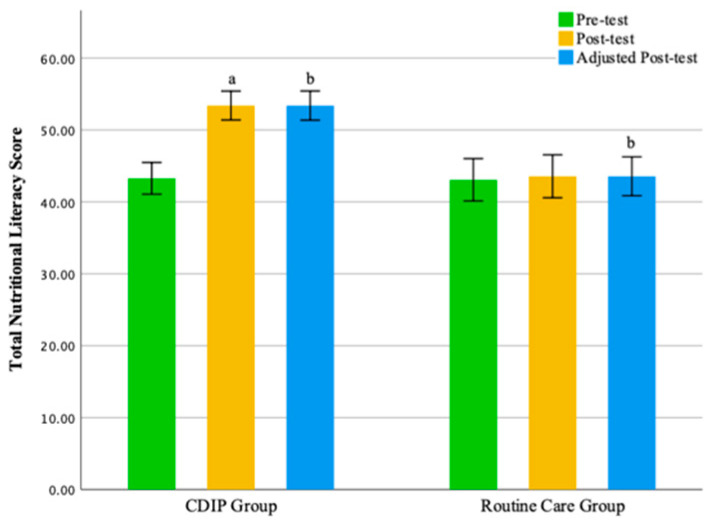
Illustrates the total nutritional literacy score before and after intervention in two groups. Note: CDIP is the Comprehensive Dietary Intervention Program, ‘a’ denotes Intra-group comparison, *p* < 0.05; ‘b’ signifies Comparison with the Control Group, *p* < 0.05.

**Figure 8:**
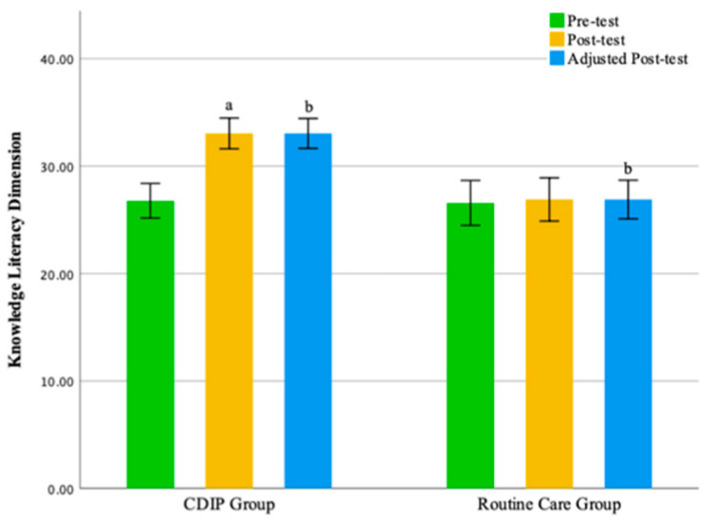
Illustrates the knowledge literacy dimension before and after intervention in two groups. Note: CDIP is the Comprehensive Dietary Intervention Program, ‘a’ denotes Intra-group comparison, *p* < 0.05; ‘b’ signifies Comparison with the Control Group, *p* < 0.05.

**Figure 9:**
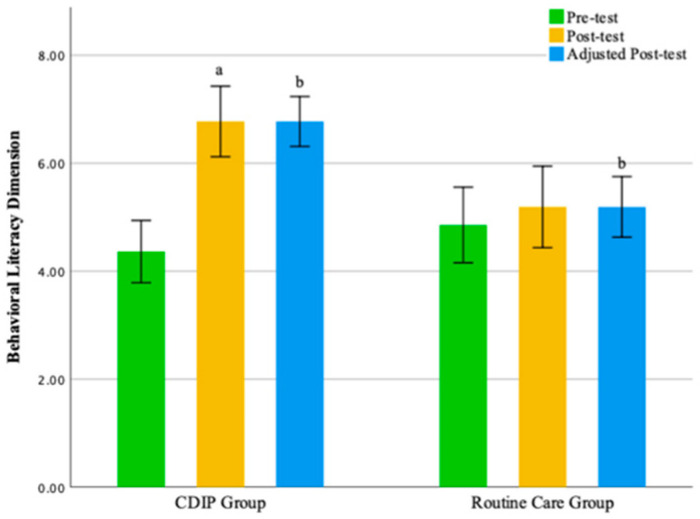
Illustrates the behavioral literacy dimension before and after intervention in two groups. Note: CDIP is the Comprehensive Dietary Intervention Program, ‘a’ denotes Intra-group comparison, *p* < 0.05; ‘b’ signifies Comparison with the Control Group, *p* < 0.05.

**Figure 10:**
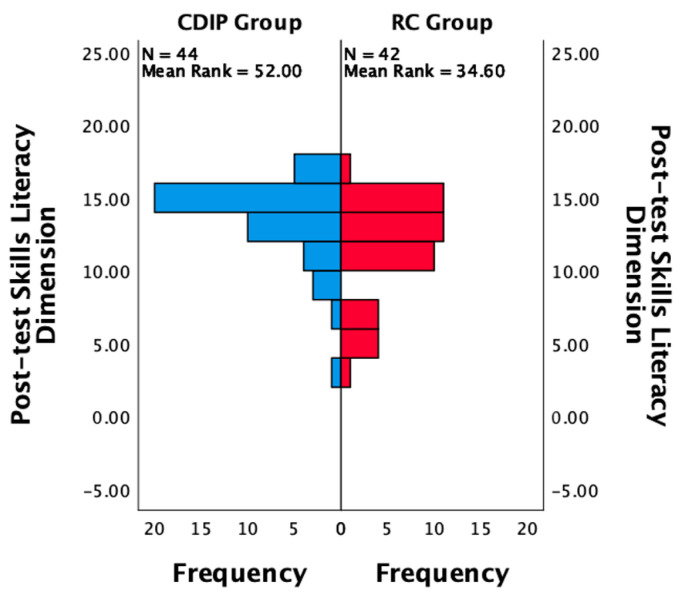
Illustrates the skills literacy dimension after intervention in two groups. Note: CDIP is the Comprehensive Dietary Intervention Program; RC is the routine care.

The results of this study suggest that, compared to the control group, CDIP intervention contributes to an improvement in nutritional literacy among pregnant women. Detailed results can be found in Table 8 and Table 9 and Figure 7, Figure 8, Figure 9 and Figure 10.

### 3.6. Impact of Intervention on Diet Quality in Urban Chinese Pregnant Women

The intra-group comparison results indicate that both groups of pregnant women, those receiving CDIP intervention and routine care, demonstrated a significantly shorter Diet Quality Distance compared to baseline (*p* < 0.05). This suggests that the overall dietary balance coefficients for both groups are closer to the balance zero point after the interventions, signifying a significant improvement.

In the CDIP group, median coefficients for nutrients other than grains and tubers, meat and poultry, and salt are significantly closer to the balance zero point compared to the baseline (*p* < 0.05), and there is an increase in the variety of food types (*p* < 0.001). In the routine care group, significant changes in median coefficients for nutrients other than animal blood or liver, vegetables, oil, and salt were observed compared to baseline (*p* < 0.05). Notably, meat and poultry, as well as eggs, shifted from a negative balance to a positive balance, with a greater distance from the zero point. The post-test median coefficient for fruits was 6, indicating a more positive deviation from the balance zero point compared to the pre-test. Seafood, soy and soy products, seaweed, nuts, dairy, and water showed shorter distances in the negative direction compared to the baseline. Additionally, there is an increase in the variety of food types (*p* < 0.001). Refer to Table 10 for details.

The inter-group comparison results, after adjusting for pre-intervention Total Diet Quality Distance levels, indicate that following an intervention, the CDIP group’s Total Diet Quality Distance is significantly shorter than the routine care group, averaging 11.49 coefficients less (95% CI: −14.730–8.242, *p* < 0.001). The post-intervention median for the variety of food types in the CDIP group is 3 higher (*p* < 0.001). In the CDIP group, the negative distance for grains and tubers is one coefficient farther compared to the routine care group (*p* = 0.042). The median coefficients for animal blood or liver, seafood, seaweed, fruits, nuts, dairy, and water are closer to the balance zero point compared to the routine care group (*p* < 0.05). There is no statistically significant difference in the post-intervention median coefficients for meat and poultry, eggs, soy and soy products, vegetables, oil, and salt between the two groups (*p* > 0.05).

**Figure 11:**
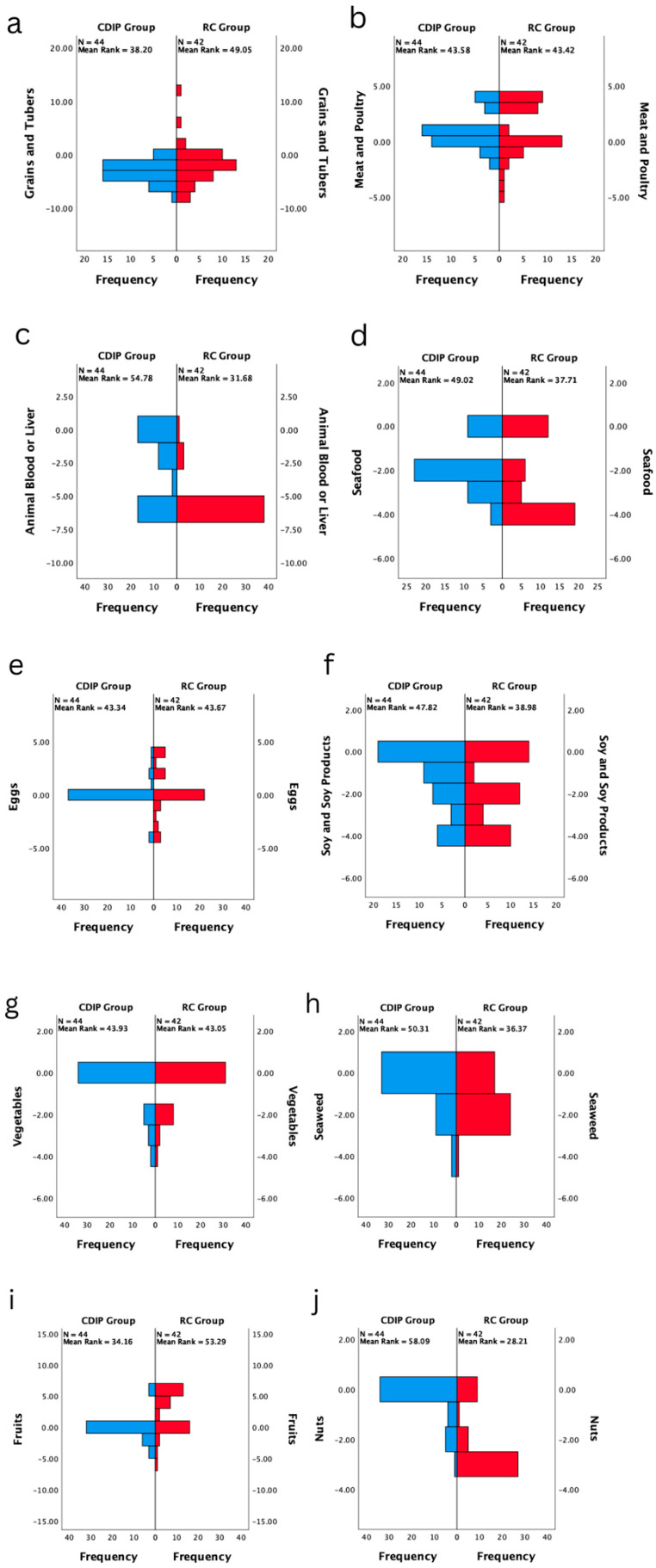

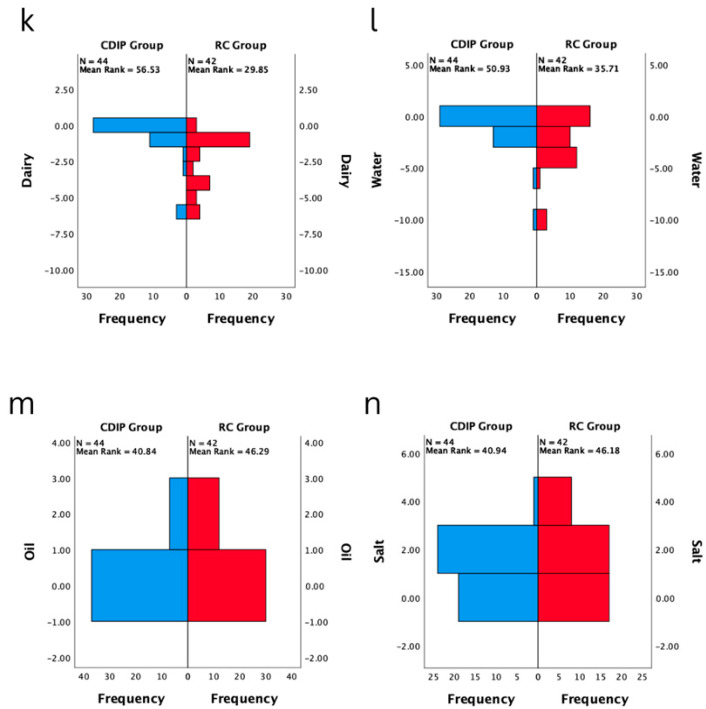
Post-test comparison of balance coefficients for various nutrients in two groups (*n* = 86). Note: (**a**) represents the comparison of frequency distributions in the dietary balance index of grains and tubers between two post-test groups; (**b**) signifies the comparison of frequency distributions in the dietary balance index of meat and poultry between two post-test groups; (**c**) denotes the comparison of frequency distributions in the dietary balance index of animal blood or liver between two post-test groups; (**d**) indicates the comparison of frequency distributions in the dietary balance index of seafood between two post-test groups; (**e**) stands for the comparison of frequency distributions in the dietary balance index of eggs between two post-test groups; (**f**) illustrates the comparison of frequency distributions in the dietary balance index of soy and soy products between two post-test groups; (**g**) showcases the comparison of frequency distributions in the dietary balance index of vegetables between two post-test groups; (**h**) highlights the comparison of frequency distributions in the dietary balance index of seaweed between two post-test groups; (**i**) focuses on the comparison of frequency distributions in the dietary balance index of fruits between two post-test groups; (**j**) portrays the comparison of frequency distributions in the dietary balance index of nuts between two post-test groups; (**k**) outlines the comparison of frequency distributions in the dietary balance index of dairy between two post-test groups; (**l**) emphasizes the comparison of frequency distributions in the dietary balance index of water between two post-test groups; (**m**) represents the comparison of frequency distributions in the dietary balance index of oil between two post-test groups; (**n**) signifies the comparison of frequency distributions in the dietary balance index of salt between two post-test groups.

**Figure 12:**
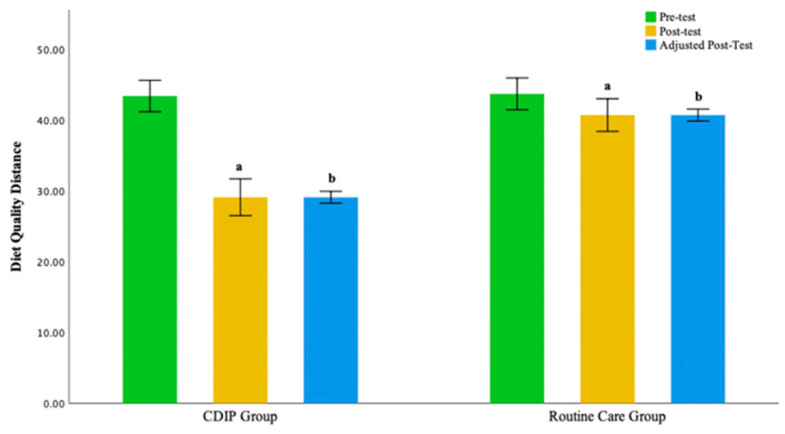
Illustrates the Diet Quality Distance before and after intervention in two groups. Note: CDIP is the Comprehensive Dietary Intervention Program, ‘a’ denotes Intra-group comparison, *p* < 0.05; ‘b’ signifies Comparison with the Control Group, *p* < 0.05.

The results of this study suggest that, compared to the control group, CDIP intervention contributes to the improvement of overall dietary quality in pregnant women. Detailed results can be found in Table 10, Table 11, Table 12 and Table 13 and Figure 11 and Figure 12.

### 3.7. Impact of Intervention on Weight Gain within 12 Weeks and Gestational Diabetes Status in Urban Chinese Pregnant Women

**Figure 13:**
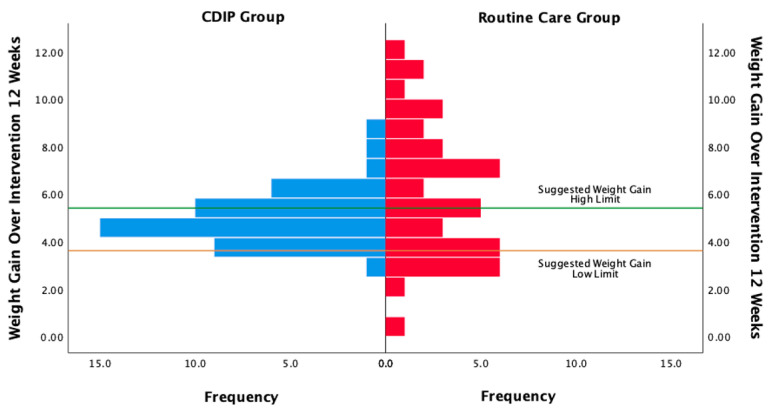
Frequency distribution of weight gain within 12 Weeks in two groups. Note: CDIP is the Comprehensive Dietary Intervention Program.

The inter-group comparison results indicate that, following the intervention, the CDIP group had a significantly lower weight gain within 12 weeks compared to the routine care group, with an average reduction of 1.01 kg (95% CI: −1.911 to −0.105, *p* = 0.029). According to the recommended standards for weight gain during the mid-pregnancy period of 12 weeks for Chinese pregnant women, which suggests a range of 3.6 to 5.4 kg, 13 individuals (29.5%) in the CDIP group and 22 individuals (52.38%) in the routine care group exceeded this standard. Additionally, two individuals (4.5%) in the CDIP group and nine individuals (37.8%) in the routine care group fell below the standard, and these differences were statistically significant (chi-square = 14.830, *p* = 0.001). Detailed frequency distributions are shown in Figure 13.

Regarding the screening and diagnosis of gestational diabetes using the OGTT test at 24 weeks of pregnancy, two individuals in the CDIP group and four individuals in the routine care group were diagnosed. However, this difference was not statistically significant. Refer to Table 14 for more details.

### 3.8. Additional Outcome Indicator Description and Analysis

Participants in the CDIP group had 641 online interactions with healthcare providers and collectively sent 1732 interactive WeChat messages over a period of twelve weeks. After watching videos, they provided 76 comments, most of which were positive. One participant expressed concerns about the reliability of the information. During the post-test interviews, healthcare providers asked participants if they encountered any significant difficulties during the engagement process. Four pregnant women mentioned that their busy work schedules left them with insufficient time to participate in midwife interactions.

Pregnant women undergoing CDIP intervention responded to 14 satisfaction-related questions in the post-test. The questionnaire had a 100% response rate. The average satisfaction scores for each question are presented in Table 15. The analysis results indicate that participants expressed high satisfaction with the CDIP intervention.

### 3.9. Comparison Analysis of Baseline General Information between Participants Lost to Follow-Up and Those Who Completed the Intervention

During the intervention, two participants were lost to follow-up, leading to incomplete data. Therefore, a comparative analysis was conducted on the baseline general information between those lost to follow-up and those who completed the intervention to determine if there was any bias. The analysis results revealed that there was no statistically significant difference in baseline general information between participants lost to follow-up and those who completed the intervention. The two groups of participants were similar in terms of age, baseline gestational weeks, family annual income, pre-pregnancy Body Mass Index, education level, cuisine preference, family function, and physical activity (*p* > 0.05). Specific results are provided in Table 16.

## 4. Discussion

The CDIP group exhibited remarkable compliance, indicating the program’s effectiveness and participant engagement. This strong adherence enhances the study’s internal validity and suggests that the intervention was well-received and valued by participants [62]. The absence of participant attrition in the CDIP group reinforces result reliability, aligning the program closely with participants’ expectations and needs [63]. The online intervention’s convenience played a pivotal role in maintaining compliance [64]. Its flexibility contributed to sustaining high levels of compliance, reducing participation barriers, and enhancing overall engagement [65]. Online platforms’ rich interactivity, enabling real-time interaction with healthcare providers, likely heightened participants’ positive experiences and sense of involvement [66,67]. Weekly interactive communication initiated by healthcare providers proved crucial in sustaining active participant engagement, maintaining interest, and facilitating questions and experience sharing [68]. This mode of communication played a key role in the successful implementation of CDIP. Future research can explore the additional functionalities and designs of online platforms to maximize the appeal and effectiveness of health behavior changes. The use of WeChat, the most widely used social platform in China, addresses the digital divide, ensuring broader population benefits [69].

In the baseline comparison of outcome measures, no significant differences were found in prenatal nutritional literacy, eating behavior, dietary quality, and weight gain during the pre-test phase, indicating the homogeneity of the study groups. However, an exploration of baseline dietary quality uncovered predominantly negative values in nutritional category coefficients, consistent with prior research on Chinese pregnant women [70]. This observation may be attributed to the influence of morning sickness, impacting dietary habits and potentially contributing to nutritional deficiencies [71]. Morning sickness, common in early pregnancy, leads to reduced appetite and altered food intake patterns, with pregnant women showing an increased intake of fruits and vegetables, possibly as a response to their comfort-inducing properties during episodes of morning sickness. This finding highlighted the need for tailored dietary guidance, acknowledging the individual variation in nutrient intake levels due to diverse dietary preferences [72]. While there were no significant differences in the median nutrient intake between the groups, the individualized nature of the nutrient intake resulted in a non-normal distribution, underscoring the importance of personalized dietary recommendations. This emphasizes the necessity for comprehensive and flexible dietary intervention programs that consider individual preferences and cultural variations to enhance overall effectiveness [73,74]. As health research evolves, future studies are expected to increasingly focus on personalized health intervention strategies, aligning with individual characteristics to better address diverse needs and expectations.

The CDIP intervention has demonstrated significant effectiveness in enhancing overall dietary behavior, particularly in promoting restrained eating. This success is attributed to a comprehensive approach that includes nutritional education, weekly weight monitoring, and reminders of weight-gain standards. Nutritional education raised the awareness of healthy pregnancy diets [75], influencing a more restrictive attitude towards food intake [76]. Weekly weight monitoring facilitates a real-time understanding of weight changes, promoting proactive restrained eating for better weight control [77]. Reminders of weight-gain standards emphasized healthy weight management goals, guiding pregnant women to consciously choose healthier eating habits [78]. However, the impact on emotional eating was not significant, possibly due to individual differences, physiological changes during pregnancy, and intervention limitations [79,80,81,82]. Additionally, the study focused on pregnant women with normal pre-pregnancy BMI, who generally may not have severe emotional eating issues [83]. This may explain why the effect of CDIP intervention on the emotional eating dimension is not significant. Since the study subjects themselves may not have significant emotional eating problems, the observed changes in this specific group may be relatively small. The lack of statistical significance in improving external eating may be attributed to CDIP’s focus on the individual level, overlooking environmental and social factors influencing external eating [84]. External eating is shaped by stimuli, social pressure, and emotional factors [85,86], which CDIP may not comprehensively address. Future research should enhance support for social and environmental factors, particularly family and societal influences, and provide comprehensive emotional health support to improve the effectiveness of external eating interventions.

CDIP intervention has demonstrated significant efficacy in improving nutritional literacy among urban Chinese pregnant women, highlighting the scientific and practical value of integrating this intervention with the health literacy framework. In comparison to the baseline, pregnant women receiving CDIP intervention showed notable improvements in overall nutritional literacy scores, knowledge literacy, behavioral literacy, and skills literacy. These findings reveal that the tailored intervention guided by the health literacy framework effectively addresses various aspects of nutritional literacy in pregnant women [87,88,89]. To further bolster the evidence of the effectiveness of CDIP intervention, inter-group comparisons were conducted across various dimensions of nutritional literacy. Findings revealed that, after adjusting for the baseline level, the Knowledge Literacy Dimension scores for the CDIP group were significantly higher than those for the routine care group, with an average difference of 6.0 points. The significance of this difference lies in the targeted elevation of pregnant women’s functional health literacy levels through CDIP intervention. By providing detailed information about prenatal diet, nutritional requirements, and food choices, CDIP encourages participants to develop a deeper understanding of their health goals, thereby enhancing their abilities to acquire information, understand and retain that information, and internalize it into applicable knowledge [90].

This study also reveals that, at the levels of skill literacy and Behavioral Literacy Dimensions, CDIP has achieved satisfactory outcomes through multidimensional intervention strategies. This indicates that CDIP has a significant impact on promoting interactive health literacy and critical health literacy among pregnant women [91]. Firstly, CDIP enhances the ability of pregnant women to disseminate dietary information within their families by training participants to conduct family dietary education themselves rather than having it directly provided by healthcare providers. This unique approach aims to empower pregnant women as leaders in health knowledge, encouraging individual learning and sharing and fostering interaction and communication. This lays the foundation for the development of interactive health literacy, enabling participants to engage in collective learning within the family and enhance health literacy through practical experiences [92]. Secondly, CDIP facilitates effective communication between pregnant women and healthcare providers through regular interactive processes, such as weekly weight-monitoring feedback and interactive dietary counseling. This interactive counseling not only provides opportunities for practical health practices but also guides pregnant women in translating theoretical knowledge into practical health decisions [93]. Through such experiential interactions, pregnant women develop critical health literacy, enabling them to accurately identify strategies that align with their individual circumstances, thereby improving the effectiveness and sustainability of health decision-making. Additionally, CDIP emphasizes interactive health literacy in family dietary education. By encouraging pregnant women to share detailed information about prenatal diet, nutritional requirements, and food choices within their families, this knowledge transfer is not only unidirectional but also involves in-depth interaction with family members, promoting closer communication [94]. This contributes to cultivating pregnant women’s understanding and analytical skills regarding different viewpoints, fostering critical thinking about health information, and, ultimately, elevating their critical health literacy [95]. In summary, CDIP, through training, regular interactive processes, and family involvement, establishes a multi-dimensional, interactive health-promotion environment. This environment enhances pregnant women’s interactive health literacy, enabling them to participate actively in health decision-making and the learning process. Additionally, it enhances critical health literacy, equipping them with the ability to discern information and make informed decisions.

The CDIP group exhibits a more diverse food intake, particularly in the supplementation of liver and animal blood, algae, and nuts, compared to the routine care group. This may reflect CDIP’s emphasis on and attention to less common dietary categories in health education. The routine care group shows relatively poorer performance in these specific dietary categories, possibly due to a lack of awareness of the importance of these nutrient sources or a deficiency in related guidance in traditional prenatal care [70,96,97]. Furthermore, CDIP’s intervention shows significant improvement in addressing the inadequate intake of seafood and dairy products. In the southern regions of China, dietary habits often lead pregnant women to insufficiently consume dairy and seafood [70,98,99]. CDIP successfully enhances the intake of these two food categories by emphasizing their nutritional importance. This highlights the positive role of CDIP’s intervention in correcting regional dietary habits and providing comprehensive nutritional support to pregnant women. On another note, CDIP emphasizes the standard intake of fruits and alerts participants to the potential adverse effects of excessive fruit consumption. This helps address the common issue of excessive fruit intake among pregnant women in mid-pregnancy [96,100]. By delivering health information on fruit consumption to participants, CDIP effectively promotes a balanced intake of fruits, contributing to preventing overconsumption and slowing the trend of excessive weight gain, thereby maintaining overall maternal health. This nuanced health education approach likely has a positive impact on adjusting dietary patterns and promoting good nutritional habits among pregnant women. However, the lack of a significant effect on salt intake in CDIP intervention results may indicate the relative stability of individual taste preferences [101]. Taste preferences are often influenced by cultural factors, personal preferences, and habits, making it challenging to change pregnant women’s preferences for salty flavors through short-term health education alone [102]. This underscores the need for a more comprehensive consideration of the complexity of taste formation in designing interventions. Future research and interventions may require a multidisciplinary approach, incorporating knowledge from psychology, sociology, and other fields, to develop more personalized and practical strategies for reducing salt intake [103].

This study reveals that participants in the routine care group experienced a dual challenge of excessive and insufficient weight gain within the first 12 weeks of mid-pregnancy, aligning with previous research on weight management in Chinese pregnant women [104]. This suggests a prevalent challenge in pregnancy-weight management in China, potentially influenced by specific cultural and lifestyle factors [36]. Furthermore, participants in the control group received general reminders during prenatal check-ups but lacked specific weight-control targets and dietary guidance. In contemporary Chinese society, diverse cultural perspectives on weight may lead to inconsistent responses among pregnant women [36]. Some may ignore the warnings due to a lack of additional information from healthcare providers or a lack of trust, resulting in uncontrolled weight gain [105]. Conversely, women who perceive weight gain as harmful to themselves or their offspring may adopt overly strict dietary measures, impeding normal weight gain [106,107]. In the absence of professional guidance, such restrictive dietary practices, rather than appropriately balancing nutrition and controlling calorie intake, may lead to nutrient deficiencies, posing significant health risks to both the pregnant woman and the fetus [108]. Evidence supporting this inference comes from the CDIP group’s intervention results. The CDIP intervention demonstrated a significant positive impact, assisting women in controlling excessive weight gain, with a greater proportion of pregnant women achieving weight gain within the industry-standard range set by the Chinese National Health Commission [109,110,111]. CDIP, by emphasizing and monitoring weight gain, provided specific and practical weekly weight-gain goals and real-time-adjusted dietary guidance. Weekly comparisons not only delivered real-time weight-management information but also motivated active participation in weight management by stressing the importance of maintaining weight gain within the standard range [112]. This personalized and frequent monitoring approach appeared to positively influence adjustments in pregnant women’s weight-gain habits.

This study analyzed the use of OGTT for gestational diabetes screening at 24 weeks. While the CDIP group had two cases, and the routine care group had four, the difference was not statistically significant. Challenges like sample size and study design may have influenced the results [113,114]. Despite the lack of statistical significance, the disparity in diagnosis rates raises practical concerns. This underscores the need for larger studies to understand the impact of dietary interventions on gestational diabetes screening and diagnosis. The findings serve as a starting point for future research and highlight challenges in clinical practice.

When conducting an in-depth analysis of participant satisfaction with the CDIP intervention, a widespread expression of high satisfaction among participants was observed. This reflects the positive evaluation of patients towards the overall intervention. Particularly noteworthy is the significant progress made in health education within the CDIP intervention. Participants gave high scores for the comprehensibility of health education (average score of 4.39 ± 0.58), indicating a high overall level of understanding of the intervention content. They perceived the provided health education materials and information as clear and understandable, contributing to an enhanced ability to make informed decisions regarding their health [45]. However, despite the generally high ratings, there is a recognition of the need to delve deeper into the underlying reasons. Potential causes for lower scores may stem from inadequate explanations of specific topics, the use of professional terminology, or information presentation methods not suitable for certain participants. For instance, the depiction of weight-monitoring charts may be challenging for some to comprehend [115]. During intervention interviews, it was discovered that one pregnant woman expressed concerns about the reliability of information. Although evidence was subsequently provided to substantiate the information’s reliability, this situation was surprising. The expressed concern highlights the aspect of patients maintaining a critical mindset in processing health information—an encouraging finding. This critical thinking not only reflects patients’ sensitivity to health information but also underscores their proactiveness in the decision-making process [116].

Simultaneously, over the 12 weeks of the intervention, participants exhibited a noticeable trend towards seeking advice on the correctness of their dietary adjustments rather than merely following the recommendations of health providers. This initiative reflects the participants’ growth in their ability to make dietary decisions, indicating that they are actively contemplating and adjusting their dietary habits. This insight has important implications for the long-term effectiveness of the intervention and the cultivation of patients’ abilities in autonomous health management [117]. Through critical thinking, patients demonstrated unique capabilities in self-health management [118]. They exhibited the ability to judiciously evaluate different dietary information, carefully considering the impact of each decision and avoiding blind conformity [118]. Critical thinking also positions patients as problem solvers, enabling them to analyze the essence of dietary challenges and actively seek practical solutions. Regarding self-monitoring, patients became more attuned to individual dietary behaviors, continuously adjusting and improving dietary plans to better meet personal health needs and goals. This comprehensive development of critical health literacy not only elevated patients’ understanding and application of health information but also made them more proactive and rational participants in their dietary management.

In the CDIP intervention, patients gave high ratings (4.70 ± 0.46) to the interactivity with health providers, indicating satisfaction with their interaction with the healthcare team. This positive feedback suggests that patients believe they can effectively engage with healthcare professionals and receive attention in problem-solving and support. However, in a more in-depth analysis, a gap between patient expectations and actual experiences of interactivity was noted. This gap might be related to the expectation that interactions should be initiated by health providers [119], possibly influenced by the reserved social culture in China [120]. Pregnant women may be hesitant to actively burden healthcare professionals but are likely to readily share their experiences once interactions commence. The intervention guidelines of this study are based on the health literacy framework, combining intervention strategies with interactive health literacy viewpoints extensively exploring potential improvements in the interaction between patients and healthcare professionals. Through an understanding of the interactive processes with pregnant women, necessary measures were derived, including actively guiding interactions, encouraging patient participation, clearly expressing openness to communication and questions, and posing questions in a gentler manner to avoid discomfort. Additionally, recommendations were made to provide regular feedback opportunities, encouraging patients to share opinions and feedback, to better understand their expectations and needs and promptly adjust interaction strategies. Implementing these measures can better meet patient expectations for interactivity, simultaneously promoting patients’ interactive health literacy, enhancing interaction effectiveness, and increasing patient satisfaction.

It is noteworthy that in the CDIP group, two individuals were diagnosed with gestational diabetes, while in the routine care group, four individuals received the same diagnosis. However, the data indicate that the difference between these two groups did not reach statistical significance. Firstly, a deeper exploration is needed into the reasons why the CDIP group did not demonstrate a superior preventive or diagnostic effect. Although it cannot be simply attributed to the ineffectiveness of the CDIP intervention, challenges such as sample size, study design, and other potential factors in academic research may have influenced the results [113,114]. In this study, the relatively small sample size may be a major contributing factor to the lack of observed significant differences. Secondly, it is crucial to explore whether these statistically non-significant differences hold potential clinical significance in actual clinical practice [121]. Despite the absence of a statistically significant difference, the disparity in the diabetes diagnosis rates between the two groups may raise concerns in practical medical settings. This underscores the distinction between statistical significance and actual clinical relevance, a common challenge in clinical research.

### Strengths and Limitations

This study’s strengths lie in its comprehensive data analysis, employing a randomized controlled trial design with a 12-week longitudinal approach. The integration of a health literacy framework adds depth to the intervention. However, limitations include a relatively small and region-specific sample size, potential biases in self-reported data, a short intervention duration, and the need for more diverse participant representation. Addressing these limitations and considering potential confounders would strengthen the study’s validity and generalizability, providing a more robust foundation for assessing the impact of the CDIP intervention on dietary and health outcomes. The study, being part of a funded program, represents the initial phase of a larger project; future studies may consider comprehensively demonstrating the effectiveness of dietary and behavioral changes in reducing risks in pathological pregnancies.

## 5. Conclusions

In conclusion, this study investigated the effectiveness of the CDIP for urban Chinese pregnant women. The results indicate positive outcomes in improving nutritional literacy, dietary quality, and restrained eating behaviors. The CDIP demonstrated success in controlling excessive weight gain and promoting a more diverse and balanced food intake. While no significant impact on gestational diabetes screening was observed, the study underscores the need for further research with larger sample sizes to explore clinical significance. Participant feedback highlighted high satisfaction and the development of critical health literacy. Despite limitations, this research contributes valuable insights for future interventions, emphasizing the importance of personalized approaches, interactivity, and long-term follow-ups for sustained impact on maternal and child health.

## Figures and Tables

**Table 1:** Comprehensive dietary intervention program structures.

Items	BCTs
Tailored information	Problem-solving Instruction on how to perform the behavior Comparative imagining of future outcomes Self-monitoring of behavior Self-monitoring of outcome(s) of behavior Conserving mental resources
Tailored communication	Social support Action planning Prompts/cues Feedback on behavior Feedback on outcome(s) of behavior
Tailored education	Behavioral practice/rehearsal

**Table 2:** General characteristics of study participants at baseline (*n* = 88).

Variable	Characteristics	CDIP Group	Routine Care Group	Statistics	*p*-Value
Education Level	Below Associate Degree	9 (20.5%)	15 (35.1%)	2.267 ^c^	0.315
	Associate Degree and Bachelor’s Degree	30 (68.2%)	26 (59.1%)		
	Bachelor’s Degree and Above	5 (11.4%)	3 (6.8%)		
Family Annual Income	<10,000 CNY	5 (11.4%)	12 (27.3%)	5.599 ^c^	0.133
	10,000 to 20,000 CNY	27 (61.4%)	20 (45.5%)		
	>20,000 to 40,000 CNY	7 (15.9%)	10 (22.7%)		
	>40,000 CNY	5 (11.4%)	2 (4.5%)		
Cuisine Preference	Hunan Cuisine	4 (9.1%)	4 (9.1%)	0.363 ^c^	0.969
	Sichuan Cuisine	5 (11.4%)	6 (13.6%)		
	Anhui Cuisine	7 (15.9%)	8 (18.2%)		
	Jiangsu Cuisine	28 (63.6%)	26 (59.1%)		
Pre-Pregnancy Body Mass Index		20.54 (19.57~22.24)	21.28 (20.23~22.60)	−1.836 ^a^	0.066
Family Function		14 (12~15)	14 (11~15)	−0.223 ^a^	0.823
Age		26.89 ± 3.47	26.14 ± 2.33	1.190 ^b^	0.237
Gestational Age at Baseline		11.89 ± 0.42	11.82 ± 0.40	0.743 ^b^	0.459
Physical Activity Level		108.69 ± 29.88	108.74 ± 27.72	−0.009 ^b^	0.993

Note: Continuous variables normally distributed are presented as mean ± standard deviation, non-normally distributed continuous variables as median (interquartile range), categorical variables as frequencies (percentages); ‘a’ indicates Mann–Whitney test; ‘b’ denotes independent *t*-test; ‘c’ signifies Fisher’s Chi-squared test.

**Table 3:** Baseline score comparison of outcome variables for study subjects (N = 88).

Variable	CDIP Group	Routine Care Group	Statistics	*p*-Value
Total Nutritional Literacy Score	43.28 ± 7.25	43.18 ± 9.24	0.056 ^b^	0.955
Knowledge Literacy Dimension	26.77 ± 5.31	26.53 ± 6.57	0.187 ^b^	0.852
Behavioral Literacy Dimension	4.36 ± 1.89	4.93 ± 2.22	−1.291 ^b^	0.200
Skills Literacy Dimension	13 (10.23~14.48)	13 (10.35~14.05)	−0.367 ^a^	0.713
Total Eating Behavior Score	86.09 ± 15.24	85.59 ± 19.12	0.136 ^b^	0.892
Restrained Eating Dimension	28.86 ± 6.82	29.20 ± 7.61	−0.221 ^b^	0.825
Emotional Eating Dimension	27.20 ± 8.96	26.11 ± 8.82	−0.575 ^b^	0.566
External Eating Dimension	30.36 ± 5.64	30.11 ± 7.05	0.184 ^b^	0.855
Weight Gain Before Pre-test	0.37 ± 2.37	−0.01 ± 3.18	0.635 ^b^	0.527
Total Diet Quality Distance	43.41 ± 7.28	43.68 ± 7.05	−0.179 ^b^	0.859
Types of Food	8.50 (8.00~9.00)	8.50 (8.00~10.00)	−0.107 ^a^	0.915
Grains and Tubers	−4.00 (−5.00~−1.25)	−4.00 (−5.00~−3.00)	−0.328 ^a^	0.743
Meat and Poultry	0 (−1.00~2.00)	−1.00 (−2.00~0)	−1.463 ^a^	0.143
Animal Blood or Liver	−6.00 (−6.00~−6.00)	−6.00 (−6.00~−6.00)	−0.575 ^a^	0.565
Seafood	−3.00 (−4.00~−1.00)	−4.00 (−4.00~−2.00)	−1.789 ^a^	0.074
Eggs	−2.00 (−4.00~0)	−2.00 (−4.00~0)	−1.235 ^a^	0.217
Soy and Soy Products	−2.00 (−3.00~−1.00)	−1.00 (−2.00~0)	−1.783 ^a^	0.075
Vegetables	0 (−2.00~0)	0 (0~0)	−1.307 ^a^	0.191
Seaweed	−2.00 (−2.00~−2.00)	−2.00 (−2.00~−2.00)	−0.633 ^a^	0.527
Fruits	5.00 (0~6.00)	5.00 (2.00~6.00)	−1.140 ^a^	0.254
Nuts	−3.00 (−3.00~0)	−2.00 (−3.00~0)	−0.600 ^a^	0.548
Dairy	−3.00 (−5.00~−1.00)	−4.00 (−5.00~−2.00)	−1.286 ^a^	0.198
Water	−3.00 (−5.00~0)	−3.00 (−5.00~−2.00)	−0.702 ^a^	0.483
Oil	0 (0~2.00)	0 (0~1.50)	−0.476 ^a^	0.634
Salt	2.00 (0~2.00)	2.00 (0~2.00)	−0.217 ^a^	0.828

Note: Continuous variables normally distributed are presented as mean ± standard deviation; non-normally distributed continuous variables as median (interquartile range); ‘a’ indicates Mann–Whitney test; ‘b’ denotes independent *t*-test.

**Table 4:** Intra-group comparison of eating behavior before and after intervention in two study groups.

Variable	Pre-Test	Post-Test	Statistics	*p*-Value
CDIP Group				
Total Eating Behavior Score	86.43 ± 14.30	88.36 ± 12.74	−1.781 ^b^	0.082
Restrained Eating Dimension	28.86 ± 6.82	31.61 ± 7.28	−4.396 ^b^	<0.001
Emotional Eating Dimension	27.20 ± 8.96	26.30 ± 8.75	2.074 ^b^	0.044
External Eating Dimension	30.36 ± 5.64	30.45 ± 4.99	−0.146 ^b^	0.885
Routine Care Group				
Total Eating Behavior Score	85.52 ± 18.03	84.78 ± 18.49	1.037 ^b^	0.306
Restrained Eating Dimension	29.14 ± 7.46	28.59 ± 8.27	1.334 ^b^	0.190
Emotional Eating Dimension	26.19 ± 9.02	26.07 ± 8.81	0.280 ^b^	0.781
External Eating Dimension	30.19 ± 7.21	30.11 ± 6.46	0.156 ^b^	0.877

Note: Continuous variables normally distributed are presented as mean ± standard deviation; “b” indicates paired *t*-test.

**Table 5:** Inter-group comparison of eating behavior after intervention in two study groups.

Variable	CDIP Group	Routine Care Group	Statistics	*p*-Value
Total Eating Behavior Score	88.00 ± 13.46	83.93 ± 18.58	2.178 ^b^	0.016
Restrained Eating Dimension	31.61 ± 7.28	28.79 ± 7.96	−4.123 ^b^	<.001
Emotional Eating Dimension	26.30 ± 8.75	26.07 ± 8.82	−1.196 ^b^	0.118
External Eating Dimension	30.45 ± 4.99	30.12 ± 6.46	0.298 ^b^	0.383

Note: Continuous variables normally distributed are presented as mean ± standard deviation; ‘b’ signifies One-way analysis of covariance.

**Table 6:** Adjusted eating behavior scores after intervention in two study groups.

Eating Behavior Scores	Mean	Standard Error	95% LCI	95% HCI
Total Eating Behavior Score				
CDIP Group	87.900	1.240	85.433	90.366
Routine Care Group	84.034	1.269	81.509	86.558
Restrained Eating Dimension				
CDIP Group	31.803	0.545	30.719	32.887
Routine Care Group	28.587	0.558	27.477	29.697
Emotional Eating Dimension				
CDIP Group	25.836	0.418	25.004	26.668
Routine Care Group	26.553	0.428	25.701	27.405
External Eating Dimension				
CDIP Group	30.392	0.486	29.425	31.359
Routine Care Group	30.185	0.497	29.195	31.174

Note: LCL stands for Lower Confidence Limit, and HCL stands for Upper Confidence Limit.

**Table 7:** Intra-group comparison of nutrition literacy before and after intervention in two study groups.

Variable	Pre-Test	Post-Test	Statistics	*p*-Value
CDIP Group				
Total Nutritional Literacy Score	43.28 ± 7.25	53.39 ± 6.60	−22.293 ^b^	<0.001
Knowledge Literacy Dimension	26.77 ± 5.31	33.05 ± 4.70	−17.029 ^b^	<0.001
Behavioral Literacy Dimension	4.36 ± 1.89	6.77 ± 2.15	−13.188 ^b^	<0.001
Skills Literacy Dimension	13.00 (10.23~14.48)	14.35 (12.75~15.55)	4.660 ^a^	<0.001
Routine Care Group				
Total Nutritional Literacy Score	43.07 ± 9.45	43.55 ± 9.58	−1.173 ^b^	0.248
Knowledge Literacy Dimension	26.57 ± 6.71	26.89 ± 6.46	−0.935 ^b^	0.355
Behavioral Literacy Dimension	4.86 ± 2.25	5.19 ± 2.42	−1.103 ^b^	0.277
Skills Literacy Dimension	13.00 (10.30~15.04)	12.30 (10.45~14.10)	−1.350 ^a^	0.177

Note: Continuous variables normally distributed are presented as mean ± standard deviation; non-normally distributed continuous variables are presented as median (interquartile range); ‘a’ indicates Paired Wilcoxon Signed Ranks Test; “b” indicates paired *t*-test.

**Table 8:** Inter-group comparison of nutrition literacy after intervention in two study groups.

Variable	CDIP Group	Routine Care Group	Statistics	*p*-Value
Total Nutritional Literacy Score	53.39 ± 6.60	43.55 ± 9.58	16.038 ^b^	<0.001
Knowledge Literacy Dimension	33.05 ± 4.70	26.89 ± 6.46	12.633 ^b^	<0.001
Behavioral Literacy Dimension	6.77 ± 2.15	5.19 ± 2.42	5.774 ^b^	<0.001
Skills Literacy Dimension	14.35 (12.75~15.55)	12.30 (10.45~14.10)	−3.234 ^a^	0.001

Note: Continuous variables normally distributed are presented as mean ± standard deviation; non-normally distributed continuous variables are presented as median (interquartile range); ‘a’ indicates Mann–Whitney test; ‘b’ signifies One-way analysis of covariance.

**Table 9:** Adjusted nutritional literacy scores after intervention in two study groups.

Variable	Mean	Standard Error	95% LCI	95% HCI
Total Nutritional Literacy Score				
CDIP Group	53.294	0.42	52.459	54.13
Routine Care Group	43.654	0.43	42.798	44.509
Knowledge Literacy Dimension				
CDIP Group	32.961	0.331	32.303	33.619
Routine Care Group	26.981	0.339	26.308	27.655
Behavioral Literacy Dimension				
CDIP Group	6.966	0.239	6.492	7.441
Routine Care Group	4.988	0.244	4.502	5.474

Note: LCL stands for Lower Confidence Limit, and HCL stands for Upper Confidence Limit.

**Table 10:** Intra-group comparison of nutrition literacy before and after intervention in CDIP group.

Variable	Pre-Test	Post-Test	Statistics	*p*-Value
CDIP Group				
Total Diet Quality Distance	43.41 ± 7.28	29.11 ± 8.52	9.403 ^b^	<0.001
Types of Food	8.50 (8.00~9.00)	12.00 (11.00~13.00)	−5.330 ^a^	<0.001
Grains and Tubers	−4.00 (−5.00~−1.25)	−4.00 (−5.00~−2.00)	−0.662 ^a^	0.508
Meat and Poultry	0 (−1.00~2.00)	1.00 (0~1.00)	−1.671 ^a^	0.095
Animal Blood or Liver	−6.00 (−6.00~−6.00)	−2.00 (−6.00~0)	−4.373 ^a^	<0.001
Seafood	−3.00 (−4.00~−1.00)	−2.00 (−3.00~−2.00)	−2.243 ^a^	0.025
Eggs	−2.00 (−4.00~0)	0 (0~0)	−3.835 ^a^	<0.001
Soy and Soy Products	−2.00 (−3.00~−1.00)	−1.00 (−2.00~0)	−2.744 ^a^	0.006
Vegetables	0 (−2.00~0)	0 (0~0)	−2.994 ^a^	0.003
Seaweed	−2.00 (−2.00~−2.00)	0 (−1.50~0)	−4.640 ^a^	<0.001
Fruits	5.00 (0~6.00)	0 (0~0)	−4.639 ^a^	<0.001
Nuts	−3.00 (−3.00~0)	0 (0~0)	−4.744 ^a^	<0.001
Dairy	−3.00 (−5.00~−1.00)	0 (−1.00~0)	−5.035 ^a^	<0.001
Water	−3.00 (−5.00~0)	0 (−3.00~0)	−3.950 ^a^	<0.001
Oil	0 (0~2.00)	0 (0~0)	−2.449 ^a^	0.014
Salt	2.00 (0~2.00)	2.00 (0~2.00)	−1.387 ^a^	0.166

Note: Continuous variables normally distributed are presented as mean ± standard deviation; non-normally distributed continuous variables are presented as median (interquartile range); ‘a’ indicates Paired Wilcoxon Signed Ranks Test”; “b” indicates paired *t*-test.

**Table 11:** Intra-group comparison of nutrition literacy before and after intervention in routine care group.

Variable	Pre-Test	Post-Test	Statistics	*p*-Value
Routine Care Group				
Total Diet Quality Distance	43.71 ± 7.22	40.71 ± 7.39	2.721 ^b^	0.010
Types of Food	8.50 (8.00~10.00)	11.00 (9.00~11.70)	−4.526 ^a^	<0.001
Grains and Tubers	−4.00 (−5.00~−3.00)	−0.75 (−3.00~0.70)	−2.596 ^a^	0.009
Meat and Poultry	−1.00 (−2.00~0)	3.00 (0~4.00)	−4.706 ^a^	<0.001
Animal Blood or Liver	−6.00 (−6.00~−6.00)	−6.00 (−6.00~−3.20)	−1.725 ^a^	0.084
Seafood	−4.00 (−4.00~−2.00)	0 (−3.00~0)	−2.357 ^a^	0.018
Eggs	−2.00 (−4.00~0)	2.00 (0~4.00)	−4.802 ^a^	<0.001
Soy and Soy Products	−1.00 (−2.00~0)	0 (−2.00~0)	−2.483 ^a^	0.013
Vegetables	0 (0~0)	0 (0~0)	−0.528 ^a^	0.598
Seaweed	−2.00 (−2.00~−2.00)	0 (−2.00~0)	−4.491 ^a^	<0.001
Fruits	5.00 (2.00~6.00)	6.00 (2.00~6.00)	−3.098 ^a^	0.002
Nuts	−2.00 (−3.00~0)	−1.75 (−3.00~0)	−2.701 ^a^	0.007
Dairy	−4.00 (−5.00~−2.00)	−1.00 (−1.00~−1.00)	−3.243 ^a^	0.001
Water	−3.00 (−5.00~−2.00)	0 (−3.00~0)	−2.114 ^a^	0.034
Oil	0 (0~1.50)	2.00 (0~2.00)	−1.414 ^a^	0.157
Salt	2.00 (0~2.00)	2.00 (2.00~4.00)	−1.414 ^a^	0.157

Note: Continuous variables normally distributed are presented as mean ± standard deviation; non-normally distributed continuous variables are presented as median (interquartile range); ‘a’ indicates Paired Wilcoxon Signed Ranks Test”; “b” indicates paired *t*-test.

**Table 12:** Inter-group comparison of dietary quality after intervention in two study groups.

Variable	CDIP Group	Routine Care Group	Statistics	*p*-Value
Total Diet Quality Distance	29.11 ± 8.52	40.71 ± 7.39	−7.043 ^b^	<0.001
Types of Food	12.00 (11.00~13.00)	9 (9.00~11.00)	−5.305 ^a^	<0.001
Grains and Tubers	−4.00 (−5.00~−2.00)	−3.00 (−4.00~−0.75)	2.031 ^a^	0.042
Meat and Poultry	1.00 (0~1.00)	0 (−0.25~3.00)	−0.031 ^a^	0.975
Animal Blood or Liver	−6.00 (−6.00~−2.00)	−6 (−6.00~−6.00)	−5.030 ^a^	<0.001
Seafood	−2.00 (−3.00~−2.00)	−3.00 (−4.00~0)	−2.181 ^a^	0.029
Eggs	0 (0~0)	0 (0~2.00)	0.074 ^a^	0.941
Soy and Soy Products	−1 (−2.00~0)	−2 (−3.25~0)	−1.708 ^a^	0.088
Vegetables	0 (0~0)	0 (−2.00~0)	−0.218 ^a^	0.827
Seaweed	0 (−1.50~0)	−2.00 (−2.00~0)	−2.994 ^a^	0.003
Fruits	0 (0~0)	2.00 (0~6.00)	3.925 ^a^	<0.001
Nuts	0 (0~0)	−3.00 (−3~−1.75)	−6.056 ^a^	<0.001
Dairy	0 (−1.00~0)	−1.00 (−4.00~−1.00)	−5.195 ^a^	<0.001
Water	0 (−3.00~0)	−3.00 (−5.00~0)	−3.083 ^a^	0.002
Oil	0 (0~0)	0 (0~2.00)	−1.407 ^a^	0.160
Salt	2.00 (0~2.00)	2.00 (0~2.00)	1.075 ^a^	0.282

Note: Continuous variables normally distributed are presented as mean ± standard deviation; non-normally distributed continuous variables are presented as median (interquartile range); ‘a’ indicates Mann–Whitney test; ‘b’ signifies One-way analysis of covariance.

**Table 13:** Adjusted Total Diet Quality Distance after intervention in two study groups.

Total Diet Quality Distance	Mean	Standard Error	95% LCI	95% HCI
CDIP Group	29.169	1.140	26.903	31.436
Routine Care Group	40.656	1.166	38.336	42.976

Note: LCL stands for Lower Confidence Limit, and HCL stands for Upper Confidence Limit.

**Table 14:** Weight gain within 12 weeks and gestational diabetes status in two groups.

Variable	CDIP Group	Routine Care Group	Statistics	*p*-Value
Weight Gain Over Intervention 12 Weeks	4.97 ± 1.33	5.98 ± 2.78	−2.220 ^a^	0.014
Gestational Diabetes Mellitus	2 (4.5%)	4 (9.5%)	0.428 ^b^	0.316

Note: Continuous variables normally distributed are presented as mean ± standard deviation; categorical variables are presented as frequencies (percentages); ‘a’ denotes independent *t*-test; ‘b’ signifies Fisher’s Chi-squared test.

**Table 15:** Satisfaction of pregnant women in the Comprehensive Dietary Intervention Program group with the intervention.

Item	Mean ± Std. Deviation
Health Education Theme Setting	4.68 ± 0.47
Health Education Comprehensibility	4.39 ± 0.58
Availability of Health Education Manuals and Video Materials	4.59 ± 0.50
Online and Offline Delivery Methods	4.64 ± 0.49
Program Flexibility	4.55 ± 0.50
Intervention Dosage	4.64 ± 0.49
Communication Methods and Language	4.73 ± 0.50
Interactivity of Health Providers	4.70 ± 0.46
Professionalism of Health Providers	4.68 ± 0.47
Supportive Role of Health Providers	4.68 ± 0.47
Positive Impact on Dietary Behaviors	4.59 ± 0.50
Positive Impact on Weight Control	4.57 ± 0.50
Generalizability	4.55 ± 0.55
Overall Satisfaction	4.70 ± 0.46

**Table 16:** Comparison of baseline general information between lost-to-follow-up and not-lost-to-follow-up study subjects (*n* = 88).

Variable	Characteristics	CDIP Group	Routine Care Group	Statistics	*p*-Value
Education Level	Below Associate Degree	24 (27.9%)	0 (0)	−1.494 ^c^	0.135
	Associate Degree and Bachelor’s Degree	54 (62.8%)	2 (100.0%)		
	Bachelor’s Degree and Above	8 (9.3%)	0 (0)		
Family Annual Income	<10,000 CNY	17 (19.8%)	0 (0)	2.128 ^c^	0.718
	10,000 to 20,000 CNY	46 (53.5%)	1 (50.0%)		
	20,000 to 40,000 CNY	19 (22.1%)	1 (50.0%)		
	>40,000 CNY	4 (4.6%)	0 (0)		
Cuisine Preference	Hunan Cuisine	8 (9.3%)	0 (0)	2.317 ^c^	0.626
	Sichuan Cuisine	11 (12.8%)	0 (0)		
	Anhui Cuisine	14 (16.3%)	1 (50%)		
	Jiangsu Cuisine	53 (61.6%)	1 (50%)		
Pre-Pregnancy Body Mass Index		201.00 (19.75~22.58)	21.23 (21.09~)	0.266 ^a^	0.790
Family Function		14 (11.75~15)	12 (11~)	−1.014 ^a^	0.310
Age		26.52 ± 2.99	26.00 ± 0	0.246 ^b^	0.807
Gestational Age at Baseline		11.85 ± 0.41	12.00 ± 0.40	−0.509 ^b^	0.612
Physical Activity Level		108.75 ± 28.98	107.00 ± 4.45	−0.080 ^b^	0.936

Note: Continuous variables normally distributed are presented as mean ± standard deviation; non-normally distributed continuous variables are presented as median (interquartile range); categorical variables are presented as frequencies (percentages); ‘a’ indicates Mann–Whitney test; ‘b’ denotes independent *t*-test; ‘c’ signifies Fisher’s Chi-squared test.

## Data Availability

The data presented in this study are available upon request from the corresponding author. The data are not publicly available due to privacy issues.

## Supplementary Materials

The following supporting information can be downloaded at: https://www.mdpi.com/article/10.3390/nu16020217/s1, Figure S1—Diet Education Booklet.
