# Supplementary material for: Effects of a Comprehensive Dietary Intervention Program, Promoting Nutrition Literacy, Eating Behavior, Dietary Quality, and Gestational Weight Gain in Chinese Urban Women with Normal Body Mass Index during Pregnancy
Source: Nutrients. 2024 Jan 10;16(2):217. doi: 10.3390/nu16020217 (PMC10820561; doi:10.3390/nu16020217)
Supplement: Supplementary file 1 [file nutrients-16-00217-s001.zip › nutrients-2781310-supplementary.pdf]

HEALTHY EATING

CDIP FOR CHINESE PREGNANT WOMEN

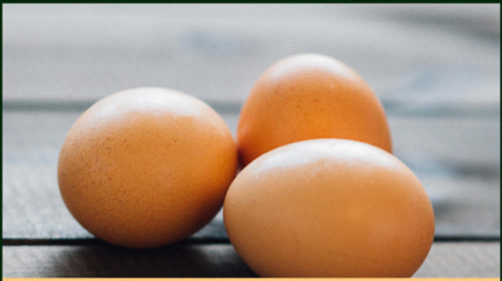

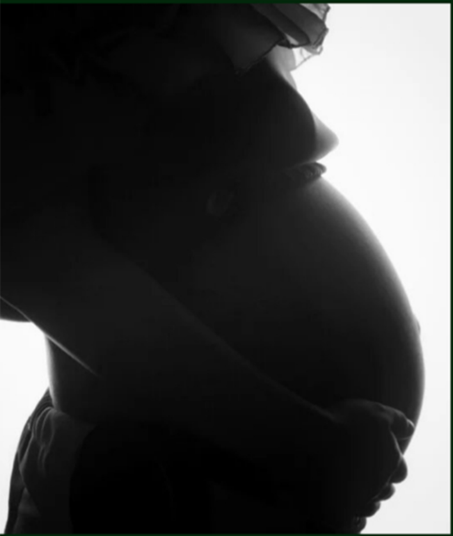

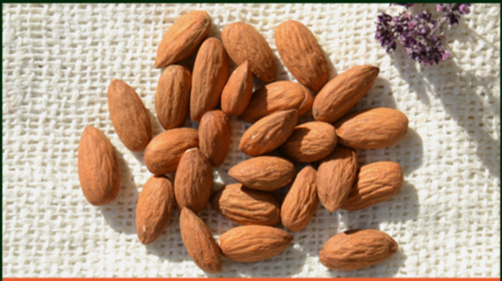

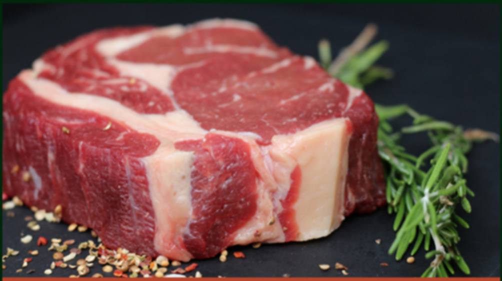

Is it necessary to manage my weight during pregnancy?

Yes, it is important to maintain a healthy weight during pregnancy. Both excessive weight gain and insufficient weight gain can pose potential risks to both the mother and the baby. Gaining too much weight during pregnancy increases the risk of developing gestational diabetes, high blood pressure, complications during labor, and difficulty losing weight after childbirth. It can also contribute to the baby growing larger, increasing the likelihood of a cesarean delivery. Conversely, inadequate weight gain during pregnancy can result in low birth weight, premature birth, and nutritional deficiencies for both the mother and the baby.

Appropriate weight gain during pregnancy depends on various factors, such as the mother's pre-pregnancy weight, overall health, and individual circumstances. The Chinese Center for Disease Control and Prevention has released a pregnancy weight gain chart for Chinese women with a pre-pregnancy normal weight, based on the weight gain guidelines during pregnancy from the US Institute of Medicine. It is recommended to aim for weight gain within the green zone indicated on the chart. You can monitor your weight on a weekly basis and compare it against this chart to assess whether your weight gain is appropriate.

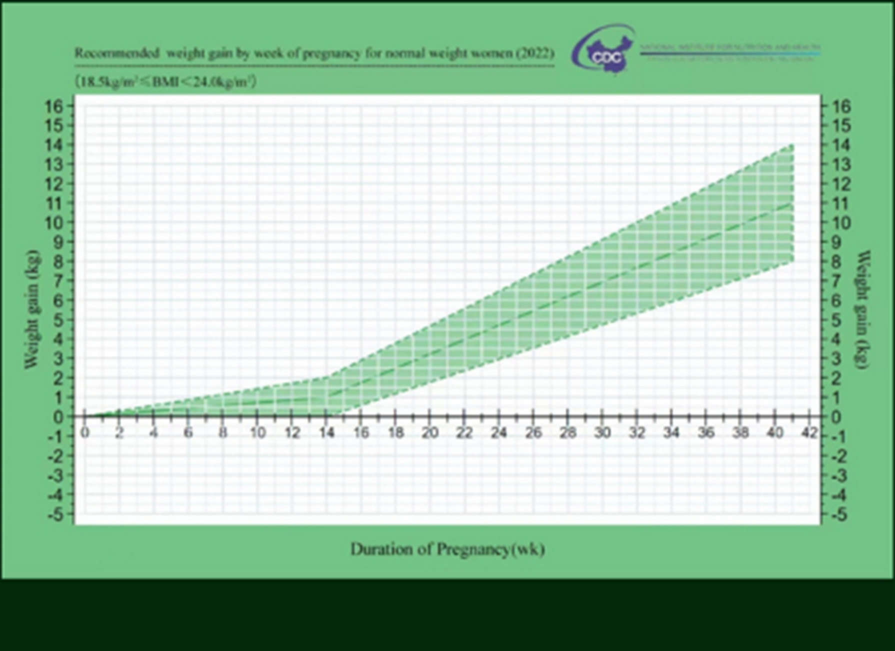

Should I "eat for two" during pregnancy?

No, there is no need to "eat for two" during pregnancy. While it is important to consume extra calories and nutrients to support the growth and development of the baby, doubling your food intake is not recommended. In fact, overeating during pregnancy can lead to excessive weight gain, which increases the risk of complications. During the first trimester, calorie needs are typically the same as before pregnancy. As you progress into the second and third trimesters, an additional 300-500 calories per day may be needed, depending on individual factors. However, it's important to focus on the quality of the calories rather than simply increasing the quantity. A balanced and nutrient-dense diet should include a variety of fruits, vegetables, whole grains, lean proteins, and healthy fats.

The Chinese Nutrition Society has issued a balanced dietary standard for pregnant women in the second trimester, taking into account the physique of Chinese pregnant women. You can evaluate whether your diet is balanced by reading the nutrient list on food labels and weighing the food you eat. Remember, the goal is to provide your body and your baby with the necessary nutrients for a healthy pregnancy, not to indulge in excessive eating. It's always recommended to consult with your healthcare provider or a registered dietitian who can provide personalized guidance on calorie intake and a balanced diet during pregnancy.

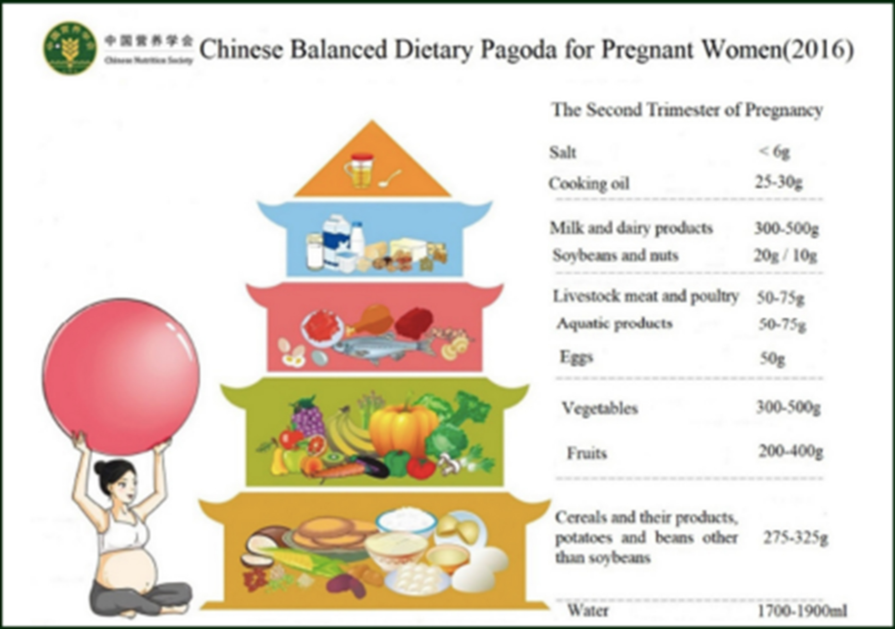

How to create a suitable meal plan according to my dietary preferences?

Various food types offer distinct nutritional content. The Chinese Center for Disease Control and Prevention has published a table presenting the nutritional content of common foods in China. You can utilize this table to substitute foods with equivalent nutritional components based on your preferences. By doing so, you can calculate the appropriate food intake according to your weight gain and develop a personalized diet plan that aligns with your dietary preferences. It is advisable to consult your healthcare provider for detailed guidance whenever possible to ensure that your meal plan meets your specific needs.

NUTRIENT CONTENT OF COMMON FOODS (WHOLE GRAINS)

	Sugar(g)	Protein(g)	Fat(g)	Vitamins						Minerals				Dietary fiber(g)	Energy
				A (ug)	E(mg)	C(mg)	Folic acid (ug)	B6(mg)	B12(mg)	Calcium	Iron(mg)	Potassium(mg)	Zinc(mg)		
Rice	76.3	7.3	0.3		0.49	7.3	2.2	1.5	19.1	7	1.5	103	1.1	0.8	337
Millet	76	9.7	3.5	12	4.1		33	0.45	68.5	29	4.7	285	3.7	1.7	374
Wheat	78	12	1.5	15	0.8		7.2	0.4	18.6	16.8	2.8	133	0.7	0.2	373.5
Corn	72.2	8.5	4.3	54	2.1	9.2	17	0.35	16.7	22	1.6	244	1.5	9.8	361.5
Soy	25.3	43.2	17.5	33.2	19.2		276	0.7		367	11	1930	4.5	4.6	429.5
Green beans	58.9	22	0.7	68	15.5	3.4	121	0.7		155	6.3	1825	3.65	5	329.9
Yam	14.4	1.7		2.6	0.5	8	13	0.18		16	0.8	473	0.62	0.6	64.4
Lotus seeds	61.8	16.6	2		3.9	3.8				120	4.9	2057	2.51	2.8	331.6
Peanut	5.2	27.6	50	5.4	3.84	9.8	70.2	0.81		7.6	3.9	674	2.33	6.8	581.2
Walnut	10	13.8	59	7.6	57		87.3	0.52		72.5	2.8	467	3.52	8	626.2
Sunflower	19.4	19	48.6	1.2	24		2.67	1.8		107	7.3	615	5.2	4.4	591
Sweet potato	29.5	1.8	0.2	27	2.9	33	54	0.7		18	0.4	6.8	0.18	0.9	127
Oat	61.8	14.2	6.4	388	3.99		20.8	0.9	56.8	177	9	324	2.93	5.1	361.6
Job's tears	79.2	12.3	4.55	550	2		19.7	0.22	143	45	4.53	252	1.27	1.8	406.9

NUTRIENT CONTENT OF COMMON FOODS (VEGETABLES)

	Sugar(g)	Protein(g)	Fat(g)	Vitamins						Minerals				Dietary fiber(g)	Energy
				A (ug)	E (mg)	C(mg)	Folic acid (ug)	B6(mg)	B12	Calcium (ug)	Iron(mg)	Potassium(mg)	Zinc(mg)		
Potato	16.4	3.3	0.1	4.3	0.57	12	23.6	0.39		10	1	309	0.26	0.4	79.7
Winter melon	1.98	0.45		11.5	0.33	19.8	29.7	0.7	0.08	20	0.4	152	0.6	0.6	18.3
Chinese cabbage	2.05	1	0.08	70	0.77	7.4	74	0.15		22	1	96	0.92	1.4	13
Fungus	65.7	10.4	0.18	15.7	13.8	5.6	79.1	0.5	5.2	357	185	733	1.85	7	306
Eggplant	3	2.3	0.2	58	1.28	7.2	23	0.11		20	0.8	168	0.49	1.2	23
Green pepper	4.3	2.2	0.4	169	192	185	43.8	2.3		10.4	0.71	297.7	0.25	2.1	29.6
Pumpkin	10.3	0.6	0.1	132	0.54	5	73	0.33		13	1.1	216	0.22	0.7	44.5
Loofah	4.1	1.4	0.15	12.3	0.37	7.4	77	0.18		26	0.7	126	0.35	0.5	23.4
Momordica charantia	3.2	0.8	0.1	9.6	1.3	113	77	0.11		3.5	1.1	179	0.6	1.2	16.9
Cucumber	3.1	0.9	0.2	22	0.91	15	27	0.9		15	0.4	107	0.39	0.6	13.8
Lily	28.1	4.1	0.2		0.9	7.8	68.2	0.35		8.1	2.3	786	3.7	5.3	131
Bamboo shoots	6.2	4	0.1	3.2	1.8	7	50	0.26		30.2	4.2	432	0.85	0.9	41.7
Celery	1.4	1.6		7.2	1.1	29	33	0.24		91	10.3	123	0.6	0.4	12
Onion	8	1.8		2.9	0.38	6.3	21	0.92		40	1.8	162	0.77	0.8	39
Spinach	2.8	2.1	0.2	22	1.9	39	120	0.84		22	1.4	152	0.6	1.4	21

NUTRIENT CONTENT OF COMMON FOODS (VEGETABLES)

	Sugar(g)	Protein(g)	Fat(g)	Vitamins						Minerals				Dietary fiber(g)	Energy
				A（ug）	E(mg)	C(mg)	Folic acid（ug）	B6(mg)	B12	Calcium（ug）	iron(mg)	Potassium(mg)	Zinc(mg)		
Radish	4.6	0.8			1.3	27	59	0.18		55	0.5	187	0.6	0.4	21.6
Lotus root	17	0.9	0.1	2.6	0.88	22				27	6.3	450	0.56	0.48	72.5
Bean sprouts	7	11.4	2.1	3.84	1.34	17	48.2	0.14		52	1.8	150	0.9	1	92.5
Lettuce	2.3	0.6	0.1	22	0.5	3.8	131	0.12		7	2	302	0.6	0.8	12.5
Water spinach	4.6	2.4	0.2	217	2.1	28	113	0.35		108	1.4	250	0.52	1.6	29.8
Tomato	3.6	0.75	0.35	88.7	0.52	7.6	27.3	0.13		8	0.4	250	0.28	0.2	20.6
day lily	62.4	14.1	1.2	297	7.3	17	42	0.15		785	9.3	543	4.22	8.7	316.8
Green beans	5.6	2.2	0.2	92	0.96	7.38	42.6	0.08		47	3.7	183	0.71	1.8	33
Carrot	8.3	0.7	0.3	830	1.1	35	22	0.33		73	10.6	198	0.37	1.3	38.7
Chinese chives	4.1	2.4	0.5	1223	6.5	39		0.7		56	1.6	311	1.6	1.6	30.5
Zizania	9.8	2.9	0.3	4.2	1.22	6	55	0.26		4	0.7	230	0.6	2.5	53.5
Taro	19.7	2.3	0.1	21.4	1.28	7.5	44.1	0.37		19	3.9	322	0.72	1.2	88.9
Coriander	7.2	1.9	0.3	38.8	1.6	41	22	0.09	1.32	170	5.6	593	0.65	3.7	39.1
Garlic	8.1	0.8	0.2	55	0.99	32.7				18	1	207	0.7	1.3	37.4
Green onions	4.1	1.2	0.3	17.8	0.42	10.5	60.7	0.38		15.9	1.34	194	1.76	1.7	23.9
Ginger	11.7	1.4	1.4	27.1	0.34	5.07	7.62	0.24		47	7	400	0.51	2.3	66

NUTRIENT CONTENT OF COMMON FOOD (FRUIT)

	Sugar(g)	Protein(g)	Fat(g)	Vitamins						Minerals				Meal fiber(g)	Energy
				A（ug）	E(mg)	C(mg)	Folic acid（ug）	B6(mg)	B12	Calcium（ug）	Iron(mg)	Potassium(mg)	Zinc(mg)		
Apple	14.8	0.4	0.5	99.2	1.82	6	6.07	0.09		12.7	0.63	3.1	0.13	0.3	65.3
Pear	14.2	0.1	0.1	97.2	1.52	5.6	8.3	0.09		5	0.2	118	0.4	2.2	58
Peach	11.1	0.8	0.1	2.39	0.92	6	4.32	0.08		8	0.81	151	0.32	0.6	48.5
Plum	8.8	0.7	0.25	23.7	0.81	5.4	43	0.06	2.95	7.6	0.73	152	0.22	0.65	40.3
Persimmon	14.6	0.4	0.15	21.4	1.3	4.5	21	0.11		147	0.8	157	0.13	1.6	61.4
Tangerine	12.1	1	0.3	63.3	1.67	42	21.9	0.06		60	1.05	138	0.29	1.7	55.1
Grape	10.9	0.6	0.5	4.2	0.52	6.7	5.1	0.11		15	0.5	135	0.1	1.6	50.5
Banana	23	1.3	0.2	58.2	0.28	11	20.1	0.44		8	0.3	325	0.24	0.6	99
Jujube	28	2.45	0.4	2.31	0.22	437	132	0.19		71.2	2.4	261.5	1.71	2.32	125.5
Mango	6.9	0.6	0.2	1320	1.34	27.3	87	0.21		206	4.3	145	0.15	1.3	31.8
Watermelon	4.2	1.3		173	0.16	3	2.87	0.12		0.6	0.17	134	0.07	0.3	22
Strawberry	4.9	1.3	2.1	1.83	0.51	51	99	0.19		25	1.75	182	0.23	1.4	43.7
Pineapple	9	0.4	0.3	31.2		36	15.2	0.13		16.3	1.02	154	0.17	0.3	40.3
Lemon	4.9	1.1	1.2	3.6	2.08	22	37	0.19		112	1.28	201	0.93	1.4	34.8
Cantaloupe	7.5	0.6	0.2	146	0.53	36.7	28.6	0.35		5.8	0.9	182	0.52	0.25	34.2
Kiwi	13	0.9	1.5	58.8	1.26	85	39	0.37		56.1	0.9	10.3	0.44	2.1	69.1
Pawpaw	5.9	0.53	0.17	138	0.37	47.6	43.2	0.03		16.4	0.7	18.5	0.36	0.65	27.3

NUTRIENT CONTENT OF COMMON FOODS (MEAT, EGGS, AQUATIC PRODUCTS AND OTHERS)

	Sugar(g)	Protein(g)	Fat(g)	Vitamins						Minerals				Cholesterol(mg)	Energy
				A(ug)	E(mg)	C(mg)	Folic acid (ug)	B6(mg)	B12(ug)	Calcium(ug)	Iron(mg)	Potassium(mg)	Zinc(mg)		
Pork	3.4	20.5	5.3	14.7	0.2	1.24	0.89	0.45	0.36	8	2.3	350	2.95	69	142.3
Pork liver	14.2	12.2	1.3	10479	0.78	31.5	997	0.76	53.7	13	23	321	3.97	309	117.3
Beef	2.6	20	10.2	2.74	0.37		7.28	0.37	1.02	7	0.9	283	1.18	59	182.2
Mutton	0.1	20	7.3	10.4	0.42	2.51	2.89	0.24	3.46	10	2	230	7.23	95	146.1
Chicken	0.3	22.3	2.3	43.1	1.77			0.46	2.37	17	2.3	346	1.6	101	111.1
Duck	0.34	17	12	51	0.13		1.87	0.45	0.74	6	2.87	230	1.05	107	177.4
Carp	0.3	17.7	10.3	23.4	1.33		4.78	0.13	11.2	117	1.85	345	2.11	83	164.7
Crucian carp	0.1	13	1.1	33.3	0.62	1.00	13.84	0.15	5.36	54	2.5	293	3.02	124	62.3
Abalone	3.4	13.5	3.5	25.3	2.12	1.12	22.5	0.11	0.33	253	22.6	129	1.68	238	99.1
Eel	0.7	18	0.8	19.8	1.53		1.87	0.45	1.52	40.4	2.2	260	0.67	118	82.7
Turtle	1.6	16.5	0.1	100	3	2	20	0.19	1.5	107	1.4	142	5.4	95	73.3
Crab	5.9	14	1.6	147	3.01		24.7	0.46	5.3	141	0.8	243	3.54	188	94
Shrimp	0.1	16.4	1.3	19	0.75		25	0.33	2.2	66	1.33	220	2.78	195	77.8
Kelp	12.1	8	0.1	38.5	0.67		21	0.13		445	4.5	1235	0.88		81.3
Milk	4.1	3.2	3.4	18	0.34	1.37	6.73	0.08	0.41	110	0.1	118	3.47	37	59.8
Peanut oil	0.6		99		38.2					15	3.02	0.94	7.45		893.4
Honey	74.3	0.6	2.1	46.2		4.25				30.6	0.42	21.6	0.04		318.5

Do you know how to deal with food cravings during pregnancy?

The first thing to remember is that it's normal to experience food cravings during pregnancy, but it's important to prioritize eating a balanced and nutritious diet for your health and the health of your baby. Here are some suggestions based on empirical research from around the world.

Identify cravings: Recognize specific foods or types of foods that you're craving. Understanding your cravings can help you find healthier alternatives or satisfy them in moderation.
Practice moderation: It's alright to indulge in your cravings occasionally, but try to practice portion control and moderation. For example, instead of consuming an entire chocolate bar, have a small piece.
Choose healthier alternatives: Find healthier substitutes to satisfy your cravings. If you have a sweet tooth, opt for fruits or yogurt instead of sugary snacks. If you're craving something salty, choose nuts or seeds instead of chips.
Balanced meals: Ensure that your daily meals are well-balanced and provide you with essential nutrients. Including a variety of fruits, vegetables, whole grains, lean protein, and healthy fats in your meals can help reduce cravings.
Stay hydrated: Sometimes, thirst can be mistaken for hunger or cravings. Drink plenty of water throughout the day to stay hydrated and reduce unnecessary cravings.
Stay active: Engaging in regular physical activity can help distract you from food cravings and improve your overall well-being. Find activities you enjoy, such as walking, swimming, or participating in prenatal yoga.
Manage stress: Stress can trigger food cravings. Find healthy ways to manage stress, such as practicing relaxation techniques, getting enough rest, or engaging in activities that bring you joy.
Seek support: Discuss your cravings with your healthcare provider or a registered dietitian. They can provide guidance, personalized advice, and support you in making healthier choices for you and your baby's well-being.

Do you know how to monitor your nutritional status during pregnancy?

Monitoring your nutritional status during pregnancy is crucial to ensure optimal health for both you and your baby. Here are some effective ways to monitor your nutritional status:

Keep a food diary: Maintaining a food diary can be a helpful tool to track your daily food intake. You can choose to use a table or take photos of your meals. Include detailed information about the types of food you eat, portion sizes, and meal times. This allows you to assess whether your meals are providing all the essential nutrients needed for a healthy pregnancy. By keeping a record of your food intake, you can identify any nutritional gaps or areas that may need improvement.

Monitor weight gain: Regularly measuring your weight at home using a standard scale is an important aspect of monitoring your nutritional status. Record your weight on a weekly basis and share the measurements with your healthcare provider. They will provide guidance on the recommended weight gain based on your pre-pregnancy weight and individual circumstances. Significant deviations in weight gain, whether excessive or insufficient, may indicate the need to address potential underlying nutritional concerns. When measuring your weight, it is advisable to use a digital scale that provides accurate measurements. Remember to stand on the scale with both feet, wear light clothing, and measure at the same time of day for consistency.

Do you know how to have a healthy eating environment at home?

Creating a healthy eating environment at home is critical to promoting overall health and supporting healthy eating habits. Here are some tips for creating a healthy eating environment:

Communication and Education: Share your knowledge of healthy eating with those responsible for cooking at home. Discuss the importance of balanced nutrition and its benefits for everyone's health. Provides evidence-based information on the CDIP. And work with the chef on a meal plan.
Stock up on healthy options: Make sure your pantry and refrigerator are stocked with nutrient-dense options. Be prepared with a variety of fresh fruits and vegetables, whole grains, lean proteins, and healthy fats. Minimize the presence of highly processed and unhealthy foods in your home.

Find common ground: If you have conflicting dietary beliefs in your family, such as traditional Chinese taboos during pregnancy versus modern dietary views, look for common ground that respects each individual's preferences and values. Emphasize the common goal of promoting good health and explore ways to incorporate different dietary philosophies into balanced and nutritious meals. The table below is based on the taboos of cold and heat during pregnancy recorded in classical Chinese medicine books "Thousands of Gold Prescriptions". It is important to note that the principles of Chinese medicine diet, which emphasize the selection of hot and cold ingredients based on individual physique, may vary for each person and should be approached with caution. These principles are based on the concepts of yin and yang and aim to restore balance within the body. However, individual responses to different foods can vary.

Traditional Chinese Food Taboos During Pregnancy		
Activating blood: can promote blood circulation and stimulate menstruation, bleeding and miscarriage	Peach kernel	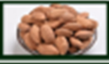
	Hawthorn	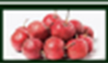
	Crab	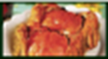
Slippery type: easy to damage the kidney qi of pregnant women, causing abnormal fetal movement , or even miscarriage	Job's tears	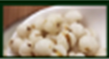
	Purslane	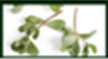
Hot food: It can cause the fetus to generate heat , get sick , and even cause bleeding and miscarriage	Cinnamon	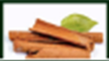
	Dried ginger	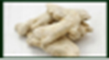
	Sparrow meat	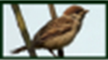
	Wine	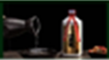
